# AAV-Tau Mediates Pyramidal Neurodegeneration by Cell-Cycle Re-Entry without Neurofibrillary Tangle Formation in Wild-Type Mice

**DOI:** 10.1371/journal.pone.0007280

**Published:** 2009-10-01

**Authors:** Tomasz Jaworski, Ilse Dewachter, Benoit Lechat, Sophie Croes, Annelies Termont, David Demedts, Peter Borghgraef, Herman Devijver, Robert K. Filipkowski, Leszek Kaczmarek, Sebastian Kügler, Fred Van Leuven

**Affiliations:** 1 Experimental Genetics Group, Department of Human Genetics, KULeuven-Campus, Leuven, Belgium; 2 Lab of Molecular Neurobiology, Nencki Institute, Warszawa, Poland; 3 Center of Molecular Physiology of the Brain (CMPB), Department of Neurology, University Medicine Göttingen, Göttingen, Germany; Mental Health Research Institute of Victoria, Australia

## Abstract

In Alzheimer's disease tauopathy is considered secondary to amyloid, and the duality obscures their relation and the definition of their respective contributions.

Transgenic mouse models do not resolve this problem conclusively, i.e. the relative hierarchy of amyloid and tau pathology depends on the actual model and the genes expressed or inactivated. Here, we approached the problem in non-transgenic models by intracerebral injection of adeno-associated viral vectors to express protein tau or amyloid precursor protein in the hippocampus in vivo. AAV-APP mutant caused neuronal accumulation of amyloid peptides, and eventually amyloid plaques at 6 months post-injection, but with only marginal hippocampal cell-death. In contrast, AAV-Tau, either wild-type or mutant P301L, provoked dramatic degeneration of pyramidal neurons in CA1/2 and cortex within weeks. Tau-mediated neurodegeneration proceeded without formation of large fibrillar tau-aggregates or tangles, but with increased expression of cell-cycle markers.

We present novel AAV-based models, which demonstrate that protein tau mediates pyramidal neurodegeneration in vivo. The data firmly support the unifying hypothesis that post-mitotic neurons are forced to re-enter the cell-cycle in primary and secondary tauopathies, including Alzheimer's disease.

## Introduction

Aggregation of hyper-phosphorylated protein tau into filaments and eventually neurofibrillary tangles (NFT) is characteristic for tauopathies, a large and diverse group of neurodegenerative disorders, including Alzheimer's disease (AD) [1]–[11]. Primary tauopathies present as clinically variable entities, e.g. Pick's disease, progressive supranuclear palsy, corticobasal degeneration and frontotemporal dementia, among others [1]. Tauopathy is defined by fibrillar and tangled aggregates of phosphorylated protein tau, which is normally a very soluble protein that binds to microtubules to secure their assembly, stability and spacing [6]–[12]. Tau3R and Tau4R isoforms have different affinity for microtubules and their relative abundance is regulated by alternative mRNA splicing [7]. Post-translational, dynamic regulation of microtubule-binding is thought to occur by phosphorylation of tau at various serine/threonine residues by various kinases, including GSK3, cdk5, and MARK, among others. In adult ageing brain, in primary tauopathies and in AD, protein tau becomes excessively phosphorylated, eventually changing its conformation to induce aggregation resulting in tauopathy [1]–[12]. Interestingly, both intronic and exonic mutations in the *MAPT* gene encoding protein tau, are dominantly associated with various tauopathies [4], [5] implying that neurotoxicity results from mutant tau protein, but as well from wild-type tau by isoform imbalances.

Alzheimer's disease (AD) is the most prominent secondary tauopathy, wherein intracellular tau inclusions combine with amyloid deposits [1]–[3]. Amyloid peptides are normal constituents in human brain at any age, stemming from amyloid precursor protein (APP) by a complex set of proteinases [13]. With ageing, amyloid peptides accumulate and aggregate, eventually becoming deposited in parenchym and vasculature, even in cognitive normal individuals as is emerging from clinical imaging studies. How and why accumulating amyloid peptides cause tauopathy, and thereby AD in some individuals and not in others, remains to be explained by genetic and environmental factors acting at the cellular, i.e. neuronal level. The relation between the two defining pathologies in AD, and their relative contribution to cognitive defects, clinical symptoms, neurodegeneration, brain atrophy and dementia remains subject to academic debate, obscures early diagnosis and hinders development of effective therapy.

Transgenic mice have been invaluable for understanding molecular mechanisms underlying amyloid peptide generation, but amyloid mice lack two major pathological features of AD, i.e. tauopathy and neuro-degeneration [14]–[18]. Tauopathy is patho-diagnostically linked to all AD-cases, including early-onset cases due to mutations in APP or presenilins that are by definition caused by amyloid overproduction. In an experimental model, absence of protein tau alleviated the cognitive defects inflicted by amyloid [18], while expressing human wild-type tau causes no or minimal tauopathy [14]–[17]. Conversely, mice expressing mutant tau associated with familial fronto-temporal dementia (FTD) recapitulate robust tauopathy [14]–[17]. Bigenic and multiple transgenic mice expressing various combinations of mutant APP and mutant tau recapitulate the combined amyloid and tau-pathology of AD, but lack neurodegeneration and brain-atrophy typical for AD [14]–[18].

Here we expressed Tau or APP, both wild-type and mutants, by adeno-associated viral vectors (AAV) injected directly into the hippocampus of wild-type mice. The observed dramatic pyramidal neuro-degeneration inflicted by wild-type Tau4R and by mutant Tau-P301L within weeks, contrasted with mutant APP that provoked amyloid pathology after 6 months but with only minor neurodegeneration. Importantly, tau-mediated neurodegeneration was not caused by fibrillar tau-aggregates. Most prominent were cell-cycle markers, indicating that degenerating neurons were attempting to re-entry the cell-cycle. The in vivo AAV-based models firmly support the unifying hypothesis that protein tau mediates neurodegeneration by forcing post-mitotic neurons to re-enter the cell-cycle in primary and secondary tauopathies.

## Results

### AAV vectors to express EGFP, APP and Tau in pyramidal neurons in vivo

Initial experiments were performed with triple mutant APP.SLA, described in the next paragraph, and mutant Tau.P301L, both packaged in AAV-vectors with hybrid serotype-1/2 [19]. Intracerebral injection of these vectors into the hippocampal complex of wild-type mice, expresses the embedded cDNA under control of the human synapsin-1 promoter, specifically in pyramidal neurons of hippocampus and cortex (Figure S1).

The generated triple mutant APP.SLA construct contained the Swedish, London and Austrian mutations that are associated with early-onset familial AD [20]–[22]. Transient expression in neuroblastoma cells demonstrated APP.SLA to produce highest levels of Aβ42 (Figure S2 A,B,C). Tau.P301L is associated with FTDP-17 and produced experimentally robust tauopathy in single and bigenic mice by us [23], [24] and others [15]–[17].

**Figure 1:**
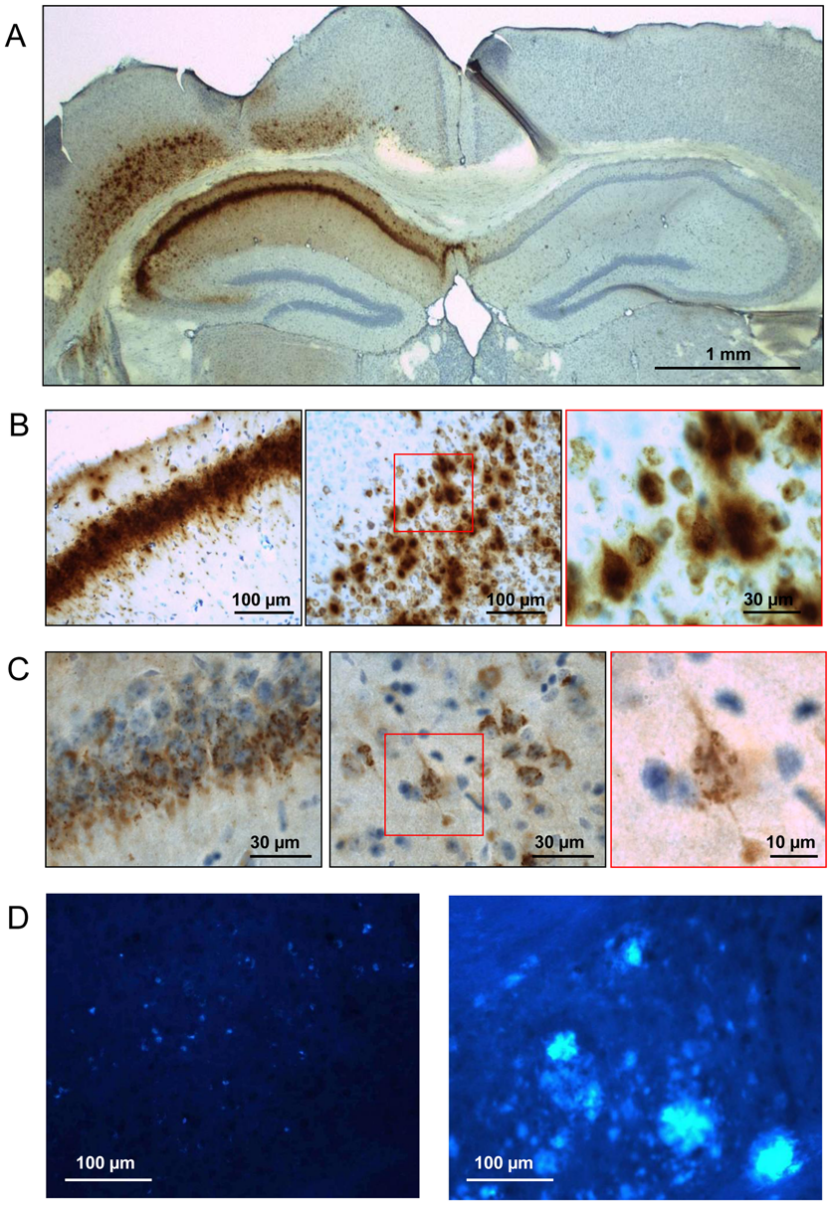
AAV-mediated expression of APP.SLA in wild-type mouse brain. Intracerebral injection of 10E8 transducing units (t.u.) AAV-APP.SLA vector in wild-type mice (n = 3) analyzed 12 weeks p.i. A, B: IHC for APP and its metabolites with Mab 6E10 on brain sections after antigen retrieval with formic acid [18]. Red square in panel B (middle) is enlarged in right panel. C: IHC for amyloid peptides with Mab 3D6; red square in middle panel is enlarged in right panel. D: histochemical staining with compound X-34 for protein aggregates [41] of AAV-APP.SLA injected mouse (left) and from an APP.V717I transgenic mouse (age 22 months) (right panel) as positive control for amyloid pathology as described [24], [29]. Scale bars panel A 1 mm, others as indicated.

Initially, brains were analyzed 12 weeks after intracerebral injection of AAV-vectors in wild-type mice (age 3–6 months). Expression of APP.SLA was pronounced in pyramidal neurons in CA and cortex (Figure 1A). Antibody 6E10 [25] revealed intense intraneuronal accumulation of APP metabolites (Figure 1B), while antibody 3D6, specific for amyloid peptides [26] showed granular intracellular amyloid deposits (Figure 1C). Amyloid plaques were not detectable at 12 weeks p.i. of APP.SLA (Figure 1D).

**Figure 2:**
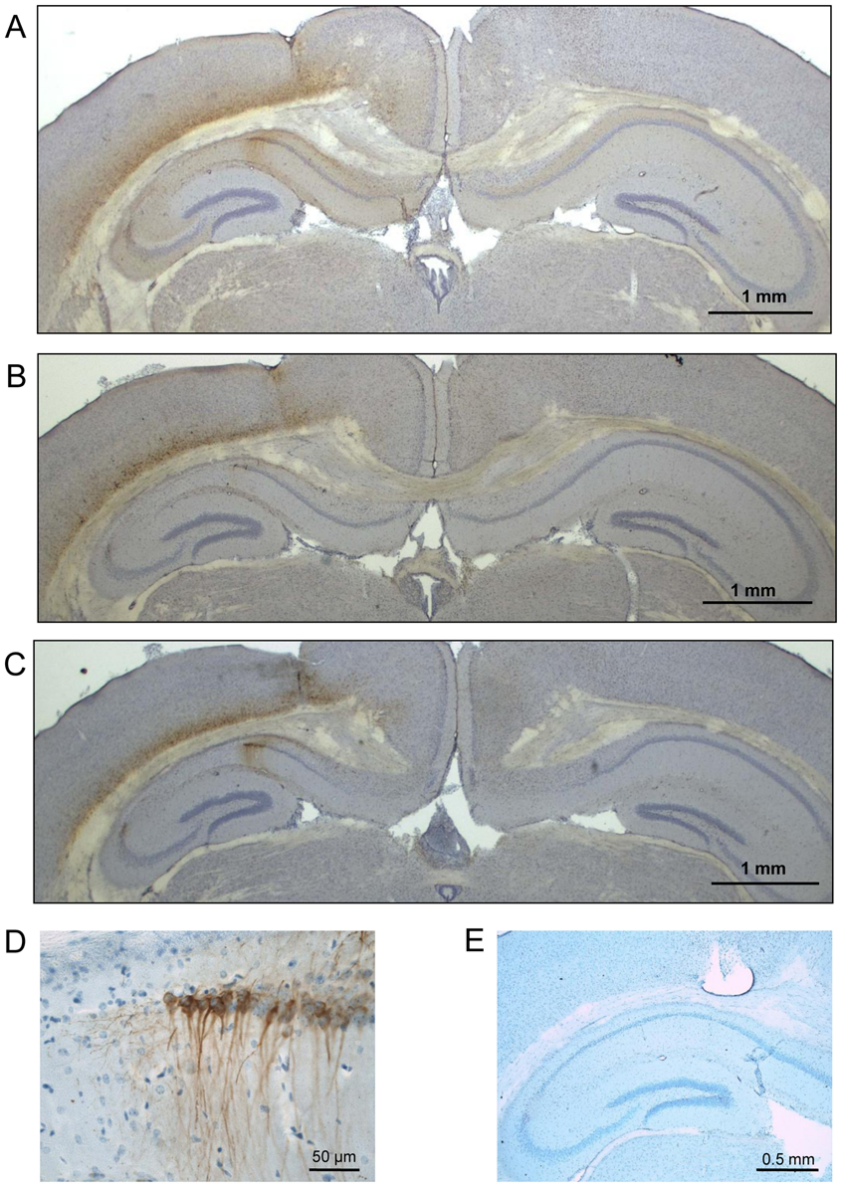
AAV-mediated expression of Tau.P301L in wild-type mouse brain. Intracerebral injection of 10E8 t.u. AAV-Tau.P301L vector in wild-type mice (n = 4) analyzed 12 weeks p.i. A: IHC for human Tau with HT7 (panel A). B, C: IHC for phospho-epitopes AT8 and AT180 respectively. D: detail of panel C at higher magnification to show pyramidal neurons on the border of CA1 and subiculum, which appear intact despite high levels of phosphorylated tau (AT180). E: IHC of AAV-Tau.P301L injected mouse at 3 weeks p.i., without primary antibody as negative control. Note the severe neurodegeneration in panels A–C and the absence of any indication of pronounced protein tau-aggregates in the degenerated regions. Scale bars A–C 1 mm, D 50 µm, E 0,5 mm.

**Figure 3:**
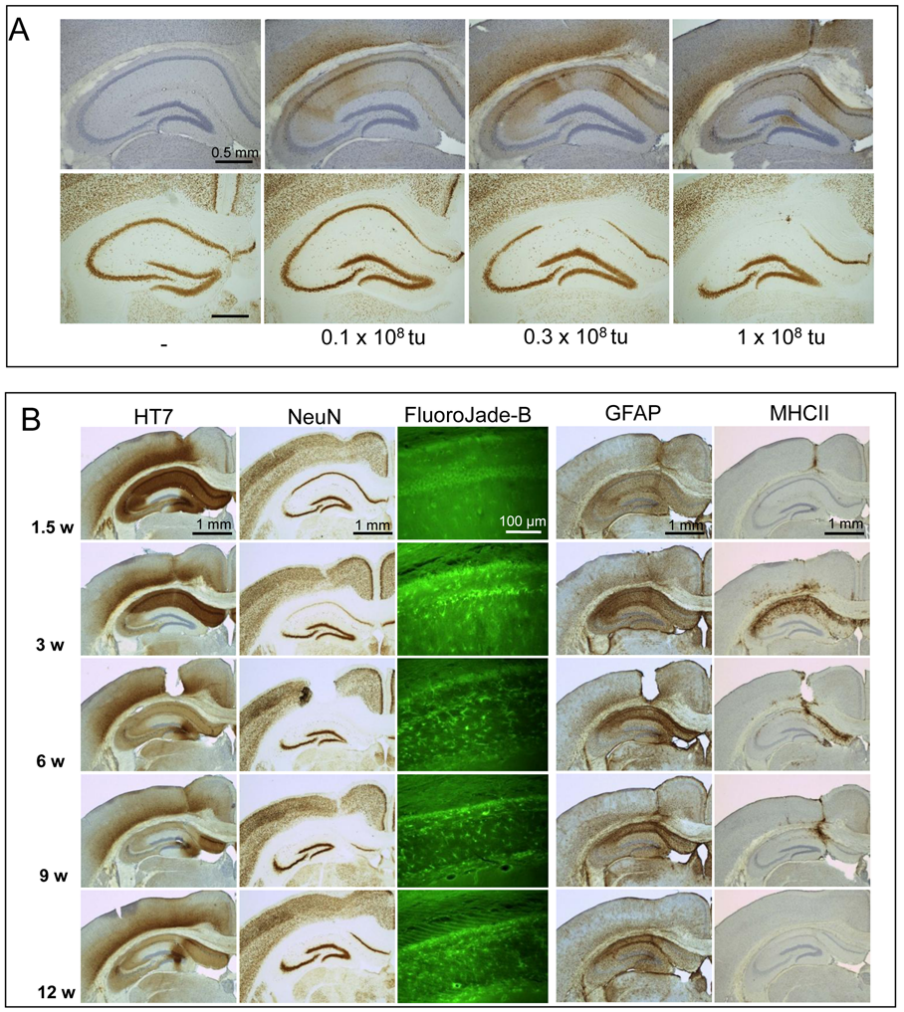
Dose-dependence and time-line of AAV-Tau.P301L mediated neurodegeneration. A. Intracerebral injection of AAV-Tau.P301L at doses indicated in wild-type mice (n = 3 per dose) analyzed at 4 weeks p.i. by IHC for total human Tau with HT7 and counterstained with hematoxylin (upper panels). IHC for NeuN visualized neuronal nuclei without counterstaining (lower panels). Scale bar 0.5 mm. Note thinning of CA1/2 already with lowest dose of APP-Tau.P301L. B. Time line of AAV-Tau.P301L mediated neurodegeneration. Intracerebral injection of 10E8 t.u. of AAV-Tau.P301L in wild-type mice analyzed at 1.5 weeks (n = 4), 3 weeks (n = 6), 6 weeks (n = 6), 9 weeks (n = 6) and 12 weeks (n = 6) p.i.. Control mice were intracerebrally injected with AAV-EGFP (10E8 t.u.) sacrificed at same time-points p.i. (all n = 4). Analysis by IHC with HT7 for total human tau, NeuN for neuronal nuclei, GFAP for astroglia and MHCII for activated microglia as indicated above the panels (scale bars 1 mm). Histological staining with FluoroJadeB for degenerating neurons and activated glia (see text for details) (scale bar 0.1 mm). Note that expression of human Tau is highest at 1.5 week p.i. and subsides later, paralleling the loss of NeuN immunoreactivity. The FJB signals in CA1 mark degenerating pyramidal neurons at 3 weeks p.i. but changes to an astroglial pattern at later time-points (see text for details). Note that at 3 weeks p.i. the loss of neurons and FJB positive signals in CA concurs with intense microgliosis, which subsides completely at 12 weeks p.i. while astrogliosis is much less specific in time and spatial distribution (see text for details).

In sharp contrast, the intracerebral injection of AAV-Tau.P301L presented at first a very surprising outcome at 12 weeks p.i., because expression of human tau was detected in cortex but was hardly detectable in the hippocampus, i.e. where the virus was injected (Figure 2 A–C). Counterstaining for nuclei and in depth analysis resolved the apparent contradiction: expression of human protein Tau was low in pyramidal neurons in the hippocampus because of the nearly complete loss of pyramidal neurons in CA1/2 (Figure 2 A–C; compare injected to contra-lateral hemisphere). The dramatic nearly complete elimination of pyramidal CA neurons was confirmed by Nissl staining (data not shown) and by IHC for NeuN as marker for neuronal nuclei (Figure 3A, lower panels).

Human protein tau was more clearly expressed in cortical neurons, as well as in apparently intact hippocampal neurons that were located adjacent to the degenerated CA regions (Figure 2 A,C,D; Figure S4B). These remaining pyramidal neurons expressed typical phospho-epitopes of tau, i.e. AT8 (pS202/pT205), AT180 (pT231), AT270 (pT181) (Figure 2, Figure S4). Importantly, the CA1/2 regions that were devoid of neurons following AAV-Tau.P301L injection did not contain any immunoreactive remains of tau-aggregates or ghost tangles (Figure 2, Figure 3A upper panel, Figure S4B).

### Tau-mediated neurodegeneration is rapid and closely associated with microgliosis

Lower doses of AAV-Tau-P301L resulted in less neurodegeneration with marked thinning of CA1/2 in injected mice (Figure 3A), coinciding spatially with expression of protein tau. The relation was actually inverse: more extensive neuron-loss contrasted with less human tau (Figure 3A), which is attributed to diminished or abolished protein synthesis in degenerating neurons.

Temporal progression of neurodegeneration, analyzed at 1.5, 3, 6, 9 and 12 weeks p.i. of AAV-Tau.P301L was evident in CA2 already at 1.5 weeks p.i., progressing to CA1 at 3 weeks p.i. and evolving into nearly complete pyramidal neuron-loss at 6–12 weeks p.i. (Figure 2A–C, Figure 3). At the later time-points neuro-degeneration was extensive also in the cortex (Figure 3B, panels marked NeuN).

FluoroJadeB (FJB) [27] strongly labeled degenerating neurons in CA2 at 1.5 weeks (not shown) and in CA1 at 3 weeks p.i. of AAV-Tau.P301L injected mice (Figure 3B, middle panels). At later time-points, neuronal FJB staining decreased in parallel with NeuN (Figure 3B), while reaction was also noted with micro- and astroglia, that were clearly activated and indicative of inflammation (Figure 3B). FJB is hereby confirmed as convenient, but not specific marker for degenerating neurons, as observed also in our inducible p25 mice, a model for hippocampal sclerosis [28].

Inflammation was confirmed by IHC for activated microglia and astroglia (Figure 3B). Microgliosis was transient and most intense at the time-points of onset and of active neurodegeneration, while fading later to even disappear completely at 12 weeks p.i. (Figure 3B, utmost right panels marked MHCII). Conversely, some astrogliosis was evident throughout the observation period (Figure 3B, panels marked GFAP). Parallel control experiments with intracerebral injection of AAV-EGFP in age- and sex-matched groups of wild-type mice did neither show neuronal loss nor microgliosis (Figure S1; results not shown).

We conclude that intracerebral injection of AAV-Tau.P301L, but not of AAV-APP.SLA or of AAV.EGFP, caused pyramidal neurons to degenerate in CA and cortex. Moreover, microgliosis is closely linked to Tau-mediated neurodegeneration, but not to amyloid production, nor to reaction to viral particles or proteins.

### Biochemical analysis of levels of expression and tau-aggregation

Biochemical analysis was performed in parallel, on four separate cohorts of wild-type mice injected intracerebrally with AAV-APP.SLA or AAV-Tau.P301L, analyzed at 1.5 weeks p.i. which marks the beginning intense neurodegeneration in AAV-Tau.P301L mice (Figure 3B). We analyzed hippocampal extracts, and not total brain extracts to avoid dilution by regions that were not transduced (Figure S3).

**Figure 4:**
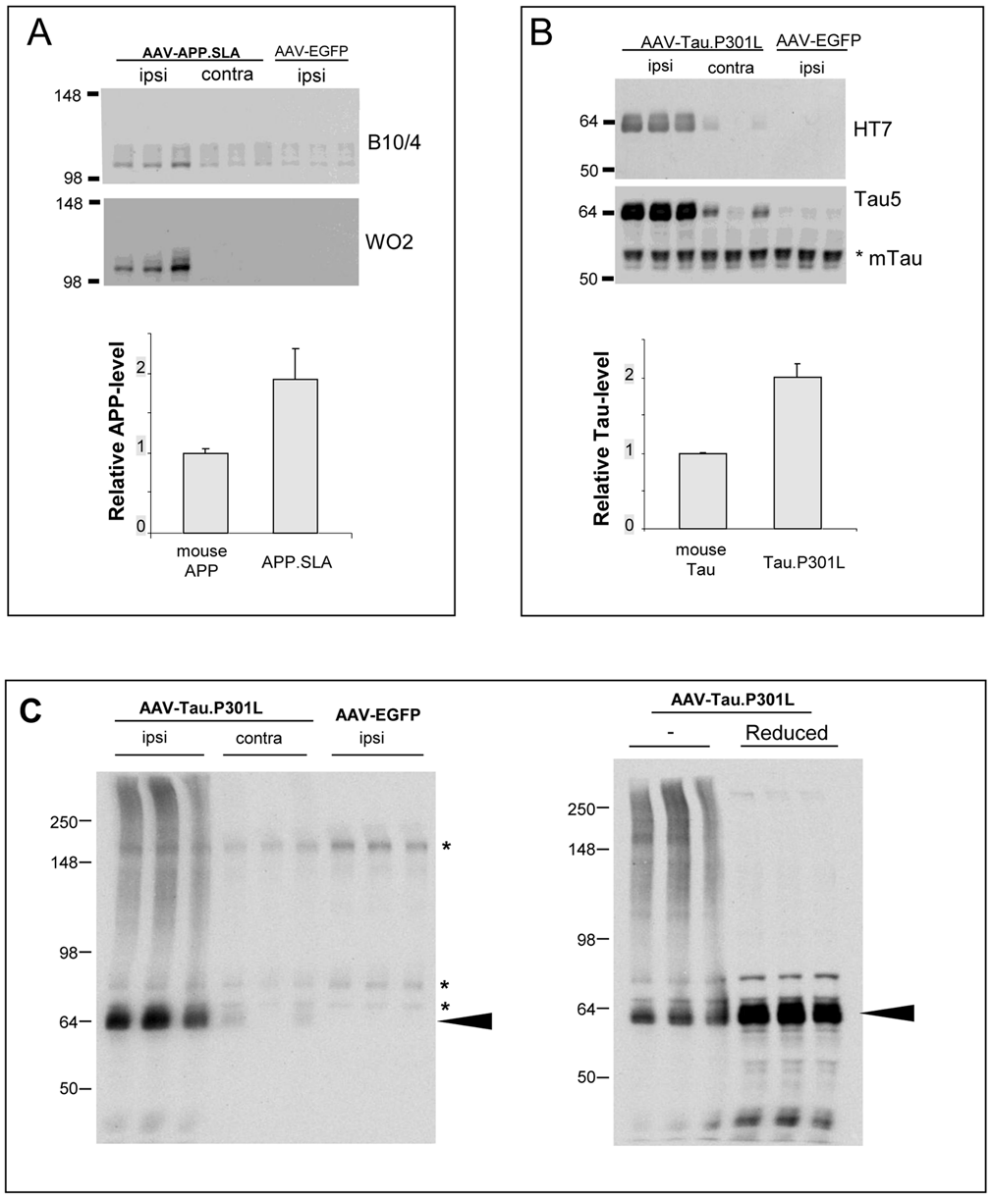
Protein levels of APP.SLA and Tau.P301L in hippocampal extracts. Biochemical analysis by western blotting of hippocampal extracts from AAV injected mice, as indicated. A: western blotting for total human and mouse APP with antibody B10/4 and for human APP with Mab WO2 on hippocampal extracts from AAV-APP.SLA injected mice at 1.5 weeks p.i. Quantitative data are from measurements with B10/4 following densitometric scanning (mean+/−SD; n = 3). B: western blotting for total human and mouse Tau with Mab Tau5 and for human Tau with Mab HT7 on hippocampal extracts from AAV-Tau.P301L injected mice at 1.5 weeks p.i. Quantitative data are from measurements with Tau5 following densitometric scanning (mean+/−SD; n = 3). C: western blotting reveals aggregated Tau oligomers in AAV-TauP301L mice. Protein extracts from AAV-Tau.P301L and AAV-EGFP injected mice (1.5 week p.i.) were separated on 8% Tris-Glycine gel under non-reducing and under reducing conditions. When blots were probed first with the secondary antibody only, non-specific bands denoted by asterisks were also revealed. Note the smears in the non-reduced samples (see text for details).

Western blotting of hippocampal protein extracts demonstrated relative levels of APP.SLA or Tau.P301L to be about two-fold higher than endogenous murine APP or murine protein tau, respectively (Figure 4A,B respectively). We conclude that neurodegeneration inflicted by Tau.P301L was not attributable to massive over-expression. Actually, pyramidal degeneration occurred at near-physiological levels of protein tau that were similar to those in our transgenic Tau.P301L mice [23], [24], which make the AAV models even more interesting.

Biochemical evidence for tau-aggregates in the brain of AAV-Tau injected mice was deduced from Western blots following SDS-PAGE under non-reducing conditions of hippocampal protein extracts. Protein-smears in the high Mr regions reacted with antibodies specific for human Tau (Figure 4C). Smears disappeared upon disulfide bond reduction concomitant with increased monomeric ∼64 kDa Tau (Figure 4C, arrowheads).

### Wild-type Tau inflicts neurodegeneration as effectively as mutant Tau.P301L

**Figure 5:**
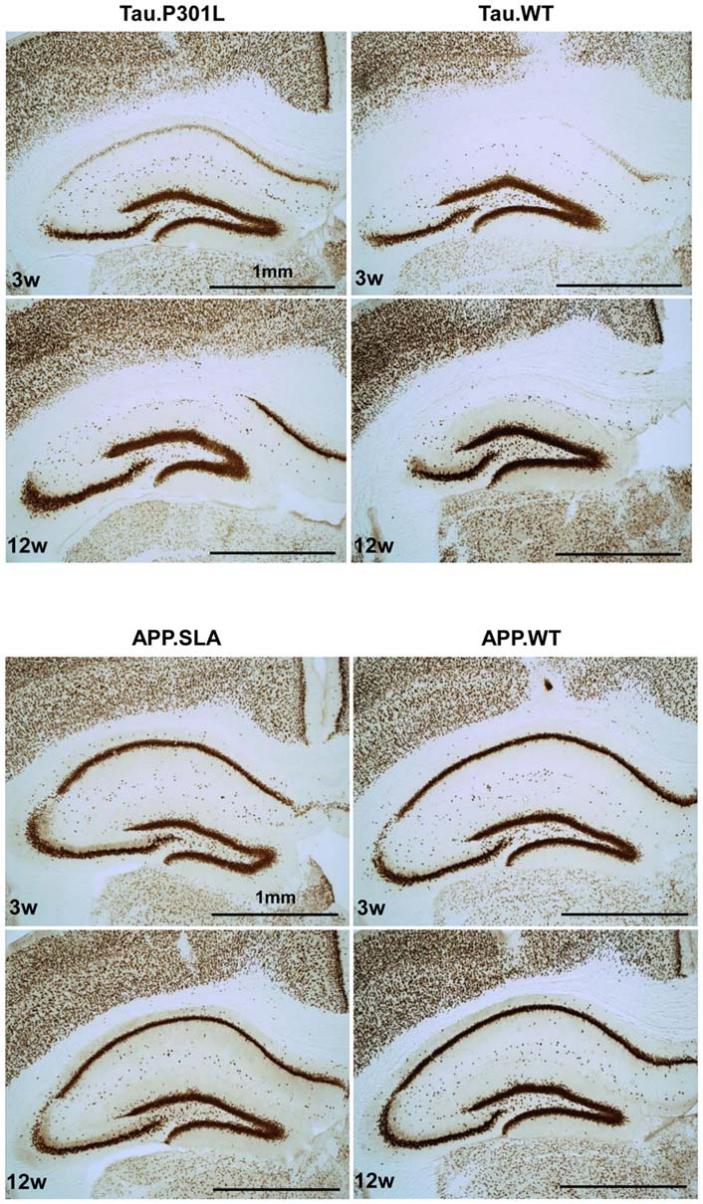
Wild-type and mutant Tau, but not APP.SLA or APP.WT, cause neurodegeneration in vivo. Intracerebral injection of 10E8 t.u. AAV-Tau.WT, AAV-Tau.P301L, AAV-APP.SLA and AAV-APP.WT vectors as indicated, in wild-type mice (n = 4) analyzed at 3 and 12 weeks p.i. by IHC for NeuN as marker for neuronal nuclei, without counterstaining. Note the dramatic neurodegeneration inflicted by wild-type and mutant Tau already at 3 weeks p.i. in contrast to AAV-APP.SLA. Scale bars 1 mm.

Additional AAV-vectors were constructed to express wild-type APP and wild-type Tau4R, in first instance as extra controls for the degeneration provoked by mutant Tau.P301L. Surprisingly, wild-type Tau4R inflicted very similar dramatic and rapid neurodegeneration in CA1/2 as mutant Tau.P301L at 3 weeks p.i. (Figure 5 upper panels, Figure S4 B). Wild-type Tau4R appeared even more deleterious at lowering protein tau levels, although these would result from more intense neurodegeneration with evident less protein synthesis (Figure 5 upper panels, Figure S4B, utmost right panels). Importantly, the findings considerably extend the potential of the paradigm, because in many tauopathies, and definitely in all AD cases, wild-type Tau4R is involved.

Conversely, wild-type APP and mutant APP.SLA compared with respect to expression of APP and the nearly unaffected appearance of pyramidal neurons in CA1/2 up to 3 months p.i.. Occasionally, NeuN appeared reduced in some AAV-APP.SLA mice (Figure 5 lower panels; results not shown). Obviously, any neuronal loss inflicted by APP.SLA is much less, or occurs much slower than the intense, rapid pyramidal neurodegeneration provoked by wild-type and mutant tau in this paradigm.

### Amyloid plaques develop in AAV-APP.SLA mice at 6 months p.i. with only marginal neuron loss

The impotency of AAV-APP.SLA in provoking neurodegeneration relative to AAV-Tau was not compensated for by time, as demonstrated in a fourth large series of experiments. We performed intracerebral injections in 4 cohorts of wild-type mice with AAV-APP.SLA or AAV-Tau.P301L, with AAV-EGFP as independent control next to the sham-injected group. All mice were analyzed for brain histology and immunohistochemistry at 6 months p.i., which is the longest time-point studied so far.

**Figure 6:**
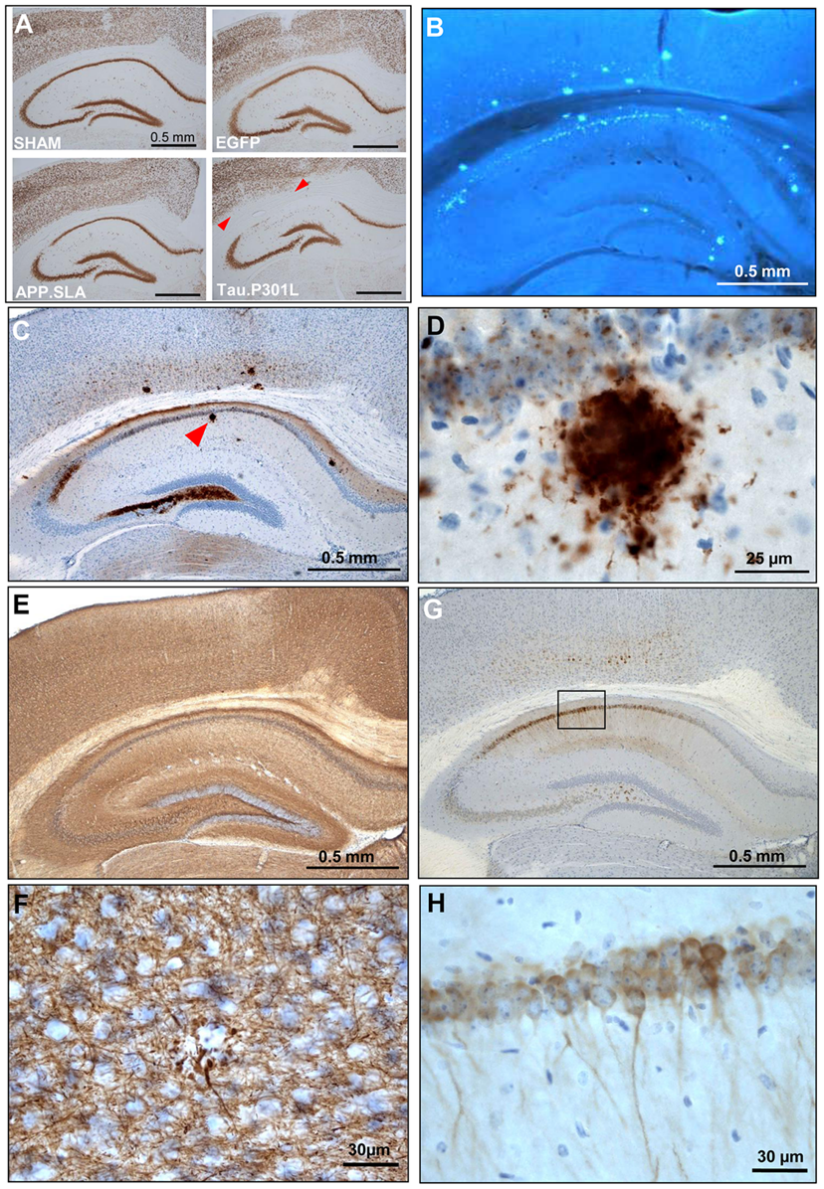
Amyloid plaques 6 months after intracerebral injection of AAV-APP.SLA. Intracerebral injection of 10E8 t.u. AAV-APP.SLA vector in wild-type mice (n = 6) analyzed 6 months p.i. A: IHC for NeuN as marker for neuronal nuclei, comparing 4 different AAV-constructs (see text for details). Note only in panel Tau.P301L the loss of pyramidal cells in CA and also in the deep cortical layers, indicated by the red arrowheads. B: X34 staining for amyloid plaques and protein aggregates [49]. C, D: IHC for amyloid peptides with Mab 3D6; plaque indicated with arrowhead enlarged in panel D. E, F: IHC with AT270 for phosphorylated endogenous mouse tau, highlights also phosphorylated tau in dystrophic neurites around amyloid plaque (panel F). AT270 was used at higher concentration than normal to reveal endogenous mouse protein Tau, which increases also the background staining (panel E, compare to Figure S4B lower panels). G, H: IHC with AT180 for phosphorylated endogenous mouse tau in CA pyramidal neurons expressing APP.SLA, with magnified detail (square in panel G in panel H). Scale bars A, B, C, E, G 0.5 mm; D 25 µm; F, H 30 µm.

In AAV-Tau.P301L mice the pyramidal neurons were again lost completely in CA and also in deep cortical layers (Figure 6A, in between red arrowheads in panel marked Tau.P301L). Conversely, in AAV-APP.SLA mice the CA regions were still largely intact at 6 months p.i., very similar to AAV-EGFP injected and sham-operated mice. Nevertheless, typical amyloid plaques were evident in hippocampus and cortex of all AAV-APP.SLA injected mice at 6 months p.i. (Figure 6B–D). This is much earlier than in APP.V717I mice and in bigenic biAT mice, which we attribute to the triple mutant APP.SLA in the current AAV model, which produces more Aβ42 than APP.V717I (Figure S2) expressed in our amyloid model [24], [29].

**Figure 7:**
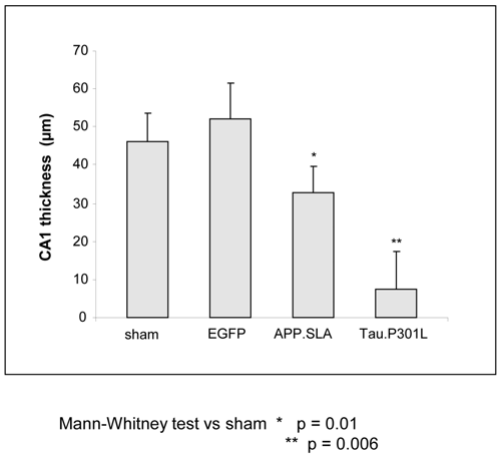
Hippocampal CA1/2 in AAV injected mice. The thickness of CA1/2 was measured at three sites along the CA region, in the four cohorts of AAV and sham-injected mice indicated (mean+/−SD; sham and APP.SLA, n = 6; EGFP and Tau.P301L, n = 5). Statistical analysis (Mann-Whitney test) versus sham, *p = 0.01, **p = 0.006.

As extra parameter for neurodegeneration, we measured the CA blade thickness in all AAV-injected mice of the four cohorts at 6 months p.i.. Relative to the nearly annihilated CA in AAV-Tau.P301L mice, these hippocampal region were hardly affected in AAV-APP.SLA mice (Figure 7). The combined data prove that mutant APP is only marginally potent and on a much longer time-scale, relative to protein Tau in provoking damage to pyramidal neurons, under very comparable experimental conditions. Moreover, IHC with AT180 demonstrated considerable tau-phosphorylation in the pyramidal neurons (Figure 6G, H) which tempts us to speculate that it is actually the beginning tauopathy that is detrimental to these neurons.

### Relation of neurodegeneration to phosphorylation and aggregation of protein tau

Intense and painstaking analysis by histological, immunohistological and biochemical methods failed to detect larger or marked aggregates of protein tau in pyramidal neurons at any time-point after injection of AAV-Tau vectors, i.e. before, during or after the documented dramatic neurodegeneration. Pathological phospho-tau epitopes were conspicuous by IHC in neurons that expressed human tau (Figure 2, Figure S4; results not shown) and were also evident in AAV-Tau mice by western blotting with specific monoclonal antibodies, e.g. AT180, AT270 and AT8 (Figure S4).

Because in biAT mice, our bigenic AD model, the amyloid pathology synergistically promotes tau phosphorylation and tauopathy in limbic regions by activating GSK3 [24], we analyzed AAV-APP.SLA mice for endogenous mouse tau phosphorylation. Specified epitopes AT270 (Figure 6E, F) and AT180 (Figure 6 G, H) were evident particularly in dystrophic neurites around plaques, as in AD patients and in our APP.V717I transgenic mice [29]. Because the AT270 antibody had to be used at higher concentrations than normal to reveal reaction with endogenous mouse protein Tau, the background staining was also increased and higher than normal (Figure 6E, compare to Figure S4B lower panels).

The biochemical and immunohistochemical data-set of phospho-epitope analysis that we have compiled is too extensive to be included here, while on the other hand it failed to reveal a direct relation of any particular phospho-epitope to the actual neurodegeneration (cfr discussion).

### Neurotoxicity of AAV-Tau depends on the microtubule binding domain

The hypothesis that microtubule binding of tau was involved in the observed neurodegeneration was supported by AAV-Tau255 that we generated to express C-terminally truncated Tau4R lacking the microtubule binding domain (Figure S5A). At 3 weeks p.i. AAV-Tau255 was efficiently expressed at similar levels as full-length Tau in limbic and cortical regions (Figure S5A). In contrast to full-length Tau, truncated Tau255 did not induce appreciable neurodegeneration, nor microgliosis (Figure S5B, C). Interestingly, Tau255 appeared more localized to neuronal somata whereas full-length Tau distributed also to somatodendritic compartments of pyramidal neurons (Figure S5D). Intriguingly, although Tau255 carried amino acid sequence 181–205 (numbering of Tau441), which upon phosphorylation constitutes the AT8 (pS202/pT205) and AT270 (pT181) epitopes, these phospho-epitopes were hardly detectable in AAV-Tau255 injected mice (Figure S5A). In contrast, phospho-epitope AT180 (pT231) is located in the same region and equally evident in pyramidal neurons in AAV-Tau255 as in AAV-Tau.4R and AAV-Tau.P301L mice (Figure S5A).

### Search for mechanisms contributing to tau-mediated neurodegeneration

The unequivocal demonstration that wild-type and mutant protein tau are equally neurotoxic for hippocampal and cortical pyramidal neurons in vivo, instigated a search for molecular markers in these novel mouse models for tau-mediated neurodegeneration. We analyzed a broad panel of markers and characteristics of degenerating neurons (Table S1, S2) based on known or suspected mechanisms that might be responsible for, or contribute to tau-mediated neurodegeneration.

Apoptotic markers were evident in AAV-Tau injected mice, albeit outside the actual degenerating regions, i.e. particularly in dentate gyrus granular cells and sparingly in CA1 adjacent to the subiculum (Figure S6A).

Beclin and Atg8/LC3 are essential regulators of autophagy that mark early and mature steps of autophagosomes [30]. The overall decrease in expression of both markers in AAV-Tau.P301L mice was evident, but also without direct temporal and spatial association with degenerating neurons (Figure S6B; results not shown). Lipofuscin was evident as intra- and extra-cellular puncta in AAV-Tau injected mice in CA pyramidal neurons and region from 3 weeks p.i. onwards, correlating with neurodegeneration and persisting in hippocampal regions after the neurons were annihilated (Figure S7; results not shown). These apparent remnant cellular debris of degenerated hippocampal neurons might support a contribution for autophagy, but also demonstrate that the complete absence of tau-aggregates in these areas is not a technical problem, and therefore conspicuous and informative.

Ultrastructurally, degenerating CA neurons presented with nuclear and cytoplasmic condensation and vacuolization, clumped chromatin and indentated or blebbing nuclear membranes (Figure S6C). These features closely resembled degenerating hippocampal neurons in our mice that model hippocampal sclerosis by inducible expression of p25, the truncated activator of cdk5 [28]. Other pathological features like tangles, spheroids and axonal dilatations were revealed by various staining methods, but only sporadically and not consistently in all AAV-Tau injected mice (Figure S6D). Neither their localization nor distribution nor abundance could explain the observed massive pyramidal cell-death.

Truncated AAV-Tau255, lacking the microtubule binding domain and failing to provoke neurodegeneration, favored the hypothesis that microtubular networks, and associated synaptic architecture, are affected which was supported by marked changes in tubulin and actin, as well as in synaptophysin (Figure S8). These features were widespread and correlated with actual degenerating neurons, although they can equally well be consequences of the inflicted pathology.

### Cell-cycle and signal transduction

Expression of cell cycle-related proteins fueled the hypothesis that post-mitotic neurons attempting to reactivate cell-cycling contribute to neurodegeneration in AD [31]–[35]. We analyzed selected markers to pinpoint an eventual role in tau-mediated neurodegeneration (Table S2).

**Figure 8:**
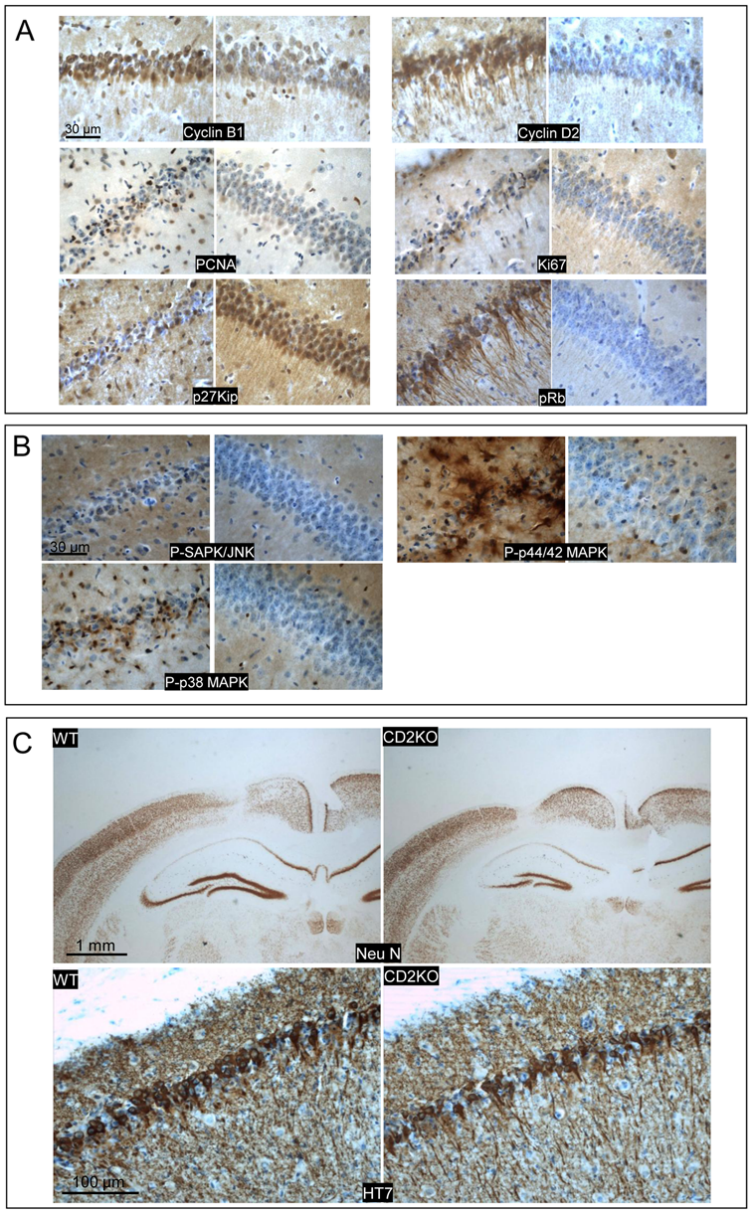
Tau-mediated neurodegeneration relates to cell cycle. A: IHC for indicated markers in brain of wild-type mice injected with 10E8 t.u. AAV-Tau.P301L analyzed 3 weeks p.i. comparing injected (left panels) to non-injected (right panels) hemispheres. Note the predominant nuclear localization of cyclinB1 and cytoplasmic expression of cyclinD2 as well as the strong phosphorylation of Retinoblastoma protein. Marker PCNA was typical for a subset of neurons in a different stage of degeneration. Scale bars 30 µm. B: IHC for active members of MAPK family: phospho-SAPK/JNK, phospho-p44/42 MAPK, phospho-p38 MAPK. Compared injected (left) to non-injected (right) hemispheres. Scale bars 30 µm. C: CyclinD2 deficient mice were injected with 10E8 t.u. AAV-Tau.P301L and analyzed 3 weeks p.i. compared to wild-type littermates (n = 4 each) by IHC for total human tau with HT7 (lower panel; scale bar 100 µm) and for NeuN as marker for neuronal nuclei (upper panel; scale bars 1 mm). Note the similar level of neurodegeneration in wild-type and CyclinD2 deficient mice injected with AAV-Tau.P301L.

Among cell-cycle and related markers, cyclinD2 and cyclinB1 were strongly up-regulated 3 weeks p.i. in degenerating neurons in AAV-Tau mice (Figure 8A) corroborated by other markers, e.g. PCNA and phosphorylated retinoblastoma protein (Figure 8A). Conversely, cell-cycle inhibitor p27KIP1 was strongly down-regulated, while markers like Ki67 and cdk2 were hardly affected by tau-mediated neurodegeneration (Figure 8A; results not shown). Effects on these and other markers were restricted to sub-regions and/or sub-sets of neurons in AAV-Tau mice, while most were unaffected in AAV-EGFP or AAV-APP.SLA injected mice (results not shown).

Ras-dependent mitogen-activated protein (MAP) kinases control cell-proliferation and stress responses. Expression of activated, phosphorylated forms of three members of the MAPK family by IHC revealed no signal for phospho-SAPK/JNK, whereas phospho-p44/42 MAPK was elevated mainly in glia cells. Only phospho-p38 MAPK was observed in a subset of degenerating neurons (Figure 8B).

The strong expression of cyclinD2, i.e. the only D-type cyclin expressed in dividing cells derived from neuronal precursors in adult hippocampus [35] prompted us to inject cyclinD2 deficient mice intracerebrally with AAV-Tau.P301L. Neurodegeneration in cyclin D2 deficient mice was, however, similar to that in wild-type littermates at 3 weeks p.i. of AAV-Tau.P301L (Figure 8C), excluding cyclinD2 on its own as decisive factor in the observed neurodegeneration.

## Discussion

Here we provide direct in vivo experimental evidence for protein tau-mediated hippocampal neuro-degeneration using intracerebral injection of specified adeno-associated viral vectors. The salient features of the model, based on extensive characterization, qualify them as innovative and unique in several aspects: protein tau at near-physiological protein levels invokes rapid and specific degeneration of pyramidal neurons in limbic regions. Moreover, wild-type Tau4R is as effective as mutant Tau.P301L which is unique, to our knowledge. Thereby AAV-tau model recapitulates, and is informative, for the majority of tauopathies that are caused by wild-type Tau4R, including all AD cases.

We discuss three important aspects that are directly concerned by the AAV-models: (i) the mechanism by which protein tau destroys pyramidal neurons; (ii) the information content of the experimental models for human disease, i.e. primary tauopathies and AD; (iii) the marked difference with classical transgenic mice that have amyloid deposits and/or tangles with minimal neurodegeneration.

### The mechanism underlying AAV-Tau neurodegeneration

Importantly, the mechanism whereby AAV-Tau inflicts neurodegeneration does not depend on the formation of large, fibrillar tau-aggregates. Conversely, surviving pyramidal neurons flanking the degenerated neurons in CA1/2, contained hyper-phosphorylated tau aggregates, but nevertheless appeared morphologically intact. This remarkable outcome corroborates our previous observation in bigenic mice with massive forebrain tauopathy, i.e. fibrillar tau is not neurotoxic per se [24]. Moreover, the combined data support the hypothesis that fibrillar tau-aggregates function as a sink, to protect neurons against smaller tau-aggregates or -oligomers that are the real neurotoxic species [24], [36]. The biochemical and physical properties of the postulated neurotoxic tau-oligomers can eventually be defined in the AAV-model, and compared to those in transgenic models. Nevertheless, that might prove a non-conclusive exercise, as discussed below. Particularly mice with inducible tau.P301L expression showed neurodegeneration and tangles but at much higher expression levels of the mutant tau.P301L [36]. To our knowledge, models based on wild-type Tau.4R have not been reported to suffer appreciable neurodegeneration in the hippocampus.

We analyzed the phosphorylation status of tau and collected extensive biochemical and immuno-histochemical data-sets on tau phospho-epitopes in the AAV models, as in our transgenic mice [23], [24]. No specific single or complex phospho-epitope on tau is identified as essential for, or directly related to the neurodegeneration. This is not unexpected, and in line with many studies on human brain and in experimental models, supporting the conviction that variable combinations of phosphorylations of protein tau underlie its neurotoxicity [1]–[11], [15]–[17], [31]. By logical extension, not a single kinase but combined actions of several kinases are to be hold responsible for phosphorylating tau to make it eventually harmful to neurons. Moreover, the sets of phospho-epitopes and of kinases most likely will vary pending the affected brain region, i.e. the disorder. Because tauopathies vary widely in their clinical, pathological and biochemical characteristics, the molecular identification of a unique, unifying neurotoxic tau-species becomes more and more unlikely.

We first and foremost consider important the only known physiological function of protein tau, i.e. binding to microtubules. If phosphorylated protein tau fails to stabilize microtubules, or affects microtubule dynamics, a dysfunctional cytoskeleton with axonal, dendritic and synaptic defects as results. The observed changes in cytoarchitecture in degenerating neurons, support the hypothesis that they contribute to the overall process. The experimental data obtained with truncated Tau255 most strongly imply that microtubule binding of protein tau is essential in inflicting neurodegeneration and involve the microtubular network as a structural and transport scaffold. The alternative explanation, i.e. that wild-type and mutant full-length Tau but not truncated Tau255 interact with cellular proteins other than microtubuli, remains open for experimental verification.

We went on to define the underlying mechanism of tau-mediated neurodegeneration by analysis of a large and wide panel of molecular targets and pathways, conform the hypothesis that neurons do not die by a single mechanism [37]. Although the outcome did not yield a single mechanism to be responsible for tau-mediated neuronal cell-death, the indications for attempted cell-cycle reentry were most marked.

### Cell cycle re-entry

In AD and unrelated tauopathies, cell-cycle events are suggested to correlate with, if not cause neuro-degeneration. Specifically, cell-cycle activation caused neurodegeneration in a Drosophila tauopathy model [38]. Here we discuss only our most marked experimental data that support the conclusion that cell-cycle re-entry is prominent in the AAV-Tau model. Supporting data and many remaining problems of this hypothesis are reviewed elsewhere [31]–[35].

Cyclins B1 and D2 were most prominent in degenerating CA1 neurons of AAV-Tau mice. Cyclins D1, D2, D3 with cdk4 and cdk6, regulate G1/S transition by releasing E2F transcription factors via increased phosphorylation of retinoblastoma protein. The latter is here also demonstrated in degenerating pyramidal neurons expressing protein Tau. Upregulation of cyclinB1 also points to the G2/M checkpoint, which depends on cdk2/cyclinB1 activity. Both cyclinD2 and cyclinB1 have been shown to mark cell-cycle events in AD brain [39]. The proliferating cell nuclear antigen (PCNA) marked a subset of CA1 pyramidal neurons, but these were, surprisingly, not marked by proliferation marker Ki67. This is particularly interesting in view of the role of PCNA in DNA repair [40].

The fact that cyclinD2 deficient mice that have problems with adult neurogenesis [35] did suffer no less neurodegeneration induced by AAV.Tau than wild-type mice, corroborates that cell cycle control, and/or induction of the post-mitotic state, is not left to one component or complex. Rather, or alternatively, the contribution of the cell-cycle to neurodegeneration would be by multi-point or by fragmented reentry attempts, whereby cyclinD2 is not the decisive factor, or is made redundant by cyclins D1 and D3 [35]. Among candidate factors that could force post-mitotic neurons to re-enter the cell-cycle are mitogenic factors from the MAPK family, e.g. p44/42 MAPK (Erk1/2), p38 MAPK and SAPK/JNK. While JNK/SAPK was not detected and p44/42 MAPK was observed in glial cells, only p38 MAPK was markedly increased in degenerating neurons in AAV-tau mice. Similarly, whereas Akt/PKB is a central regulator of metabolism, apoptosis, transcription and cell-cycle, this kinase appeared not activated in the AAV-Tau model.

From the many markers analyzed (Tables S1, S2) we conclude that the strongest indications support the hypothesis that neurons degenerate because of their attempts to re-enter the cell-cycle. Nevertheless, other markers are observed and proposed to contribute, not in the least microgliosis, as discussed below. Activated microglia are spatially and temporally closely associated with degenerating neurons in the AAV-tau models, strongly resembling the pathological characteristics of mice with hippocampal and cortical sclerosis [28].

### The relevance for human disease: tauopathies and AD

The rapid and dramatic neurodegeneration inflicted by protein tau contrasts with the minimal neurotoxic effects sorted by the mutant APP.SLA under the same experimental conditions. Only marginal neuro-degeneration resulted despite important accumulation of amyloid peptides that led even at 6 months p.i. to amyloid plaques in hippocampus and cortex. This marked dissociation in outcome by the same experimental approach, corroborates the growing awareness that neurodegeneration in AD is not mediated primarily or directly by amyloid. The essential contribution of protein tau to pyramidal cell-death is thereby joined seamlessly to the primary tauopathies. Consequently, tau pathology is essential and decisive, together with amyloid, in the overall pathogenesis of AD, notwithstanding its pathological classification as a ‘secondary tauopathy’.

We adhere the hypothesis that in AD the accumulation of amyloid is the trigger, but that protein tau executes specified neurons. The transgenic models for amyloid and tau pathology have not, however, substantiated this hypothesis to the fullest, although they have been invaluable for understanding molecular mechanisms of amyloid peptide generation, amyloid pathology, repercussions on cognition and behavior [13]–[17]. Even multigenic mice that express various combinations of mutant APP and mutant tau to recapitulate the combined amyloid and tau-pathology of AD, still lack the specific regional neurodegeneration that leads to the typical brain atrophy in AD [13]–[17].

Remarkably, transgenic mouse models now even support the reverse hypothesis, i.e. that tangle deposition is protective, similar to amyloid plaques in amyloid pathology. Both are sinks for mis-folded, aggregated, indigestible peptides or proteins. Whereas such detoxification by aggregation and deposition can be effective as a short-term strategy, it eventually becomes futile and then aggravates the pathology, particularly in humans with their long life-span. Of note, the view on tauopathy is evolving very analogous as that on amyloid pathology in AD: the emphasis shifts from large visible deposits, i.e. amyloid plaques and neurofibrillary tangles, to smaller molecular entities, i.e. amyloid- and tau-oligomers. A similar evolution is evident in other neurodegenerative disorders.

The combined transgenic and AAV-models allow us to propose confidently that the link between amyloid, tau pathology and neurodegeneration does not involve directly the formation or repercussions of amyloid plaques and neurofibrillary tangles. Rather, these are the products of dead-end escape-routes whereby cells, in the case of AD pyramidal neurons in limbic brain regions, attempt to limit the negative impact of accumulating misfolded and aggregated proteins that cannot be properly handled by normal cellular proteolytic degradation mechanisms, i.e. endosomal, lysosomal, auto-phagosomal, proteasomal, … It is then not surprising to see many of these involved in, or attained by, the amyloid or tau pathology.

Although tauopathy with tangles is and remains the post-mortem pathological hallmark that is co-diagnostic for AD, the genetic evidence of *Tau* mutations in familial primary tauopathies has promoted tauopathy to be mechanistically relevant and important. Experimental evidence in vivo that was largely lacking is convincingly provided by the current AAV-models.

### AAV versus transgenic models

A final important point concerns the question why AAV-based models are more powerful in producing neuro-degeneration than transgenic models expressing the same wild-type Tau4R isoform [41] or Tau.P301L mutant [23] at similar near-physiological levels. These transgenic models suffer axonopathy and tauopathy, respectively, but without appreciable neurodegeneration.

Although data to answer this problem do not abound, we consider as major difference the observed microgliosis that is much more intense in the AAV-Tau model than in the AAV-APP mice. Microgliosis is spatially and temporally most closely associated with degenerating neurons in the AAV-tau models. This is strongly reminiscent of our observations in inducible p25 mice that suffer a profound hippocampal and cortical sclerosis with pathological characteristics very similar to the AAV-Tau mice [28]. A recent study described wild-type tau to mediate some neurodegeneration with combined microgliosis by AAV gene-transfer [42]. Therein, degeneration of dopaminergic neurons in the substantia nigra of aged rats was also directly associated with microgliosis, lending support to our assumption that microgliosis contributes essentially to neurodegeneration. Whereas also the viral vectors used as delivery tool, can be of some importance, they are evidently not decisive because neurodegeneration is specific for AAV-tau, wild-type and mutant, and was lacking in AAV-APP and AAV-EGFP mice, observed here and as observed in other models [19], [42].

Aspects that are important to explain the apparently contradictory outcome in different models, relate to differences in time-scale and/or kinetics whereby neurons either degenerate or whereby tau aggregates and ‘sinks’ into tangles. It is evident that various post-translational modifications, e.g. phosphorylation, ubiquitinylation, glycation, O-glucosylation, … can and will contribute to either mechanism. The overall process is complex and implies enzymes, i.e. proteinases, kinases, phosphatases, … but also cellular factors like chaperones and heat-shock proteins. Their structural features and dynamic actions will tilt the balance to either slow death by progressive accumulation of aggregated, undigested or undigestible amyloid and/or protein tau, or to faster death by cell-cycle re-entry, accelerated by microglia derived pro-inflammatory neurotoxic factors [43].

Moreover, the tau-species that are responsible for aggregation and neurotoxicity are proposed to differ at the molecular level. We refer here also to a most recent report on the transmission and spreading of tauopathy in transgenic mouse brain, following intra-cerebral injection of tau-aggregates [44]. Those findings are relevant for the possible cell-to-cell spreading of tauopathy in brain and imply an extracellular route, which is to be defined for the cytoplasmic protein tau. Nevertheless, the time-scale of spreading was very slow and resulted in typical tauopathy with aggregates and tangles, while neuro-degeneration was minimal or absent [44]. Thereby, that model conforms to the tauopathy as observed in the parental tau.P301S transgenic mice that have no neurodegeneration in limbic regions.

In conclusion, we present in vivo experimental evidence for a major problem in tauopathies: effective modeling of pyramidal neurodegeneration that is mediated by protein tau.4R, which is responsible for the majority of human tauopathies, including all Alzheimer patients. We further delineate two major mechanisms that contribute to the rapid neurodegeneration mediated by AAV-Tau: attempted cell-cycle re-entry by the post-mitotic neurons, and microgliosis. We are confident that these innovative models will contribute considerably to unravel the molecular factors and mechanistic details. Importantly, the ease whereby the AAV-vectors and the models can be implemented widely in research-projects on neurodegeneration is a further strong point of this report. The wider distribution of the model, and its application for analysis of mechanisms that will untangle tau-mediated neurodegeneration, is hoped to help meet the needs of patients suffering from primary tauopathies or Alzheimer's disease.

## Materials and Methods

### Generation of AAV-vectors

Human cDNA of wild-type and mutant P301L in the longest tau isoform (Tau4R/2N) was used as described [23], [24], [41], [45]. Human APP695 cDNA was used as wild-type or as triple mutant, containing the Swedish, London and Austrian mutations (denoted APP.SLA). The mutations were introduced by site directed mutagenesis by standard techniques.

For cloning into the pAAV vectors [19] the cDNA was amplified from the plasmids by polymerase chain reaction (Phusion DNA polymerase; New England Biolabs), with primers for tau

5′- AAAAAAGGATCCACCGGTCGCCACCATGGCTGAGCCCCGCCAGGAGTTCGAAGTGATGG – 3′

5′- TTTTTTGCGGCCGCTCACAAACCCTGCTTGGCCAGGGAGGCAGACACCTCGTCAGC – 3′.

and primers for APP

5′- AAAAAAGCTAGCAAGGATCCACCGGTCGCCACCATGCTGCCCGGTTTGGCACTGCTCCTGCT - 3′

5′- TTTTTTGCGGCCGCCTAGTTCTGCATCTGCTCAAAGAACTTGTAGGTTGG – 3′

These primers contain suitable restriction sites for cloning into the pAAV-constructs. Amplified Tau cDNA was inserted into BamHI-NotI sites while amplified APP cDNA was inserted into NheI-NotI sites of pAAV. Integrity of the ITR was confirmed by restriction with SmaI and by sequencing. All molecular cloning procedures were performed in SURE bacteria (Stratagene, La Jolla, California) to minimize recombination events.

Recombinant AAV vectors of hybrid serotype 1/2 were produced essentially as described [45], [46] to express either EGFP, human Tau (WT or mutant P301L) or human APP (WT or SLA triple mutant) under control of the human synapsin1 gene promoter [19]. Vectors were purified from cell lysates by iodixanol step gradient centrifugation followed by heparin affinity chromatography. Eluted virus was dialyzed against PBS and stored in single use aliquots at −80°C. Vector genomes were titrated by quantitative PCR and purity validated by SDS-PAGE and silver staining.

### Animals and stereotaxic injection

Adult wild-type FVB/N mice were used for most studies, except for the cyclin D2 deficient mice [47] that were maintained in the C57Bl background. Intracerebral injection of viral particles in the left hemisphere of anesthetized mice (Nembutal, i.p. 0.7 mg/kg) was performed stereotactically at coordinates posterior 1.94 mm, lateral 1.4 mm, ventral 2.2 mm relative to bregma [48]. Standard injection was 2 µl of a viral suspension containing 10E8 transducing units (t.u.) using 10 µl glass syringes with a fixed needle (Hamilton, Reno, Nevada). After injection at a rate of 0.5 µl/min, the needle was left in place for 5 min before withdrawal.

All animal experiments were performed by certified researchers conforming to regional, national and European regulations concerning animal welfare and animal experimentation, authorized and supervised by the university animal welfare commission (Ethische Commissie Dierenwelzijn, KULeuven). We formally declare that we comply to the European FP7-Decision 1982/2006/EC, Article 6§1, i.e. all research activities is carried out in compliance with fundamental ethical principles and all experiments are approved and overlooked by the respective Animal Welfare Commissions.

### Immunohistochemistry

At the indicated time periods post-injection (p.i.) mice were anesthetized and perfused transcardiacally with ice-cold saline for 2 min. Brains were removed rapidly and fixed overnight in 4% paraformaldehyde for subsequent immunohistochemical analysis on free-floating coronal vibratome sections (40 µm). Primary antibodies were either affinity-purified polyclonal antibodies or mouse monoclonal antibodies that were biotinylated (Table S1). Immune reactions were developed with streptavidin-HRP complex for monoclonal antibodies or by a three-step method for polyclonal antibodies [18], [26]. Sections were counterstained with hematoxylin, except after NeuN staining, dehydrated by passage through a graded series of alcohol and xylol and mounted in DepeX for microscopic analysis.

### Biochemistry

In separate experiments, the brain of injected mice was processed for biochemistry. Following anesthesia and perfusion as above, the brain was removed, dissected into hippocampus and cortex on ice and snap-frozen in liquid nitrogen. Hippocampi were homogenized in 6 volumes of homogenization buffer, i.e. 25 mM Tris·HCl (pH 7.6), 150 mM NaCl, 1 mM EDTA, 1 mM EGTA, 20 mM sodium fluoride, 1 nM okadaic acid, 0.2 mM sodium vanadate, 1 mM phenylmethylsulfonyl fluoride, and complete protease inhibitor cocktail and stored at −20°C until analysis. For SDS-PAGE total homogenate was loaded onto 8% Tris-glycine gels (Invitrogen, Merelbeke, Belgium). Following separation, proteins were transferred to nitrocellulose membranes at 180 mA for 2.5 hr and membranes were blocked for 1 hr with 5% non-fat milk-powder in TBST before incubation with primary antibodies (Table S1) at 4°C overnight, followed by incubation with secondary antibody at RT for 1 hr. Immune-reactions were visualized (ECL, GE-Health, Amersham, UK) and signals captured on photographic film for scanning and densitometric analysis (Lab-scan and ImageQuant TL, GE-Health, Amersham, UK).

### Histology

Compound X34 is a derivative of Congo Red binding to beta-pleated proteins [49]. Sections were washed in PBS and incubated in 10 µM X34 in 40% ethanol, 50 mM Tris-HCl pH 9.5 for 10 min. Sections were rinsed in tap water, treated with 0.2% NaOH in 80% ethanol, washed in tap water and mounted on silanised glass-slides, dried at 40°C for 2 hr before mounting in Depex. FluoroJadeB was used as described [24], [28]. Sections were mounted on gelatin-coated glass-slides and sequentially treated with 1% NaOH, 80% ethanol (5 min), 70% ethanol (2 min) and water (2 min). Sections were oxidized by 0.06% KMnO4 solution, 2 min, and washed in deionized water. After incubation with FluoroJadeB (0.0004% in 0.1% acetic acid, 10 min) sections were rinsed in deionized water, dried at 50°C (10 min) and cleaned in xylol before mounting in Depex.

### Ultrastructural analysis

Anesthetized mice (Nembutal, i.p. 1.7 mg/kg) were perfused transcardiacally with ice-cold saline (2 min) followed by Karnovsky fixation for 10 min. Brains were removed and stored in Karnovsky solution at 4°C. Thick vibratome sections (300 µm) were cut and incubated with 1% osmium tetroxide (60 min) before dehydration through a graded series of ethanol and impregnation with Agar100 resin. Ultrathin sections (80 nm) were stained with uranyl acetate and lead citrate by standard procedures before analysis by transmission electron microscopy (160 kV; JEM-2100; Jeol, Tokyo, Japan).

## Supporting Information

Figure S1 AAV1/2-mediated expression of EGFP. Intracerebral injection of 10E8 t.u. AAV-EGFP in wild-type mice analyzed 12 weeks months p.i. by fluorescence microscopy for EGFP in neurons in the hippocampal formation and in layers 5 and 6 of the cortex (upper panel). Confocal microscopy of CA1 (lower left panel) and CA2 (lower right panel) pyramidal neurons with EGFP expressed in soma and apical dendrites. Scale bar 40 µm.
(8.88 MB TIF)

Figure S2 Effect of APP-mutations on generation of amyloid peptides in transfected N2a. Mouse neuroblastoma N2a cells transiently transfected with pcDNA3 vectors encoding human APP695 containing no, Swedish (K670M/N671L), London (V717I) or Austrian (T714I) mutations, alone and in the combinations indicated. Panels A:. cellular growth media were collected after 48 hours of culture, immunoprecipitated with Mab 6E10 and protein G-agarose beads before Western blotting with Mab WO2, after microwave heating of the filters, as recommended for the WO2 antibody. Panel B: Cell extracts analyzed directly by Western blotting with WO2. Panel C: acid-urea SDS-PAGE to separate amyloid peptides with synthetic amyloid peptides as standards (Aβ-mix). Quantification by densitometric scanning using synthetic peptides as standards. Note the highest ratio Aβ42/Aβ40 for APP.SLA triple mutant, which was used in the AAV-construct.
(1.40 MB TIF)

Figure S3 Distribution of human tau following AAV-Tau.P301L injection. Compilation of 40 sections (each 40 µm) spaced each about 3–4 sections apart throughout the brain of a wildtype mouse injected with 10E8 t.u. AAV-TauP301L and analyzed at 1.5 weeks p.i. for human protein tau by IHC with Mab HT7.
(10.05 MB TIF)

Figure S4 Comparison of wild-type and mutant Tau. Intracerebral injection of 10E8 t.u. of the indicated AAV-vectors in wild-type mice analyzed 1.5 week (panel A) and 3 weeks p.i. (panel B) with indicated antibodies in Western blotting and IHC, respectively. Note some minor cross-reaction of the polyclonal antibody against Tau.P301L (ref. 46) with wild-type Tau in IHC (right upper panel).
(10.17 MB TIF)

Figure S5 Protein Tau255 lacking microtubuli binding domains is not neurotoxic. Intracerebral injection of 10E8 t.u. AAV-Tau255 vector in wild-type mice (n = 8) analyzed 3 weeks p.i. A: representation of Tau.255 and Tau4R constructs and representative IHC for human Tau with HT7, AT180, AT8, AT270. Note that Tau.255 lacks phosphorylation at AT8 and AT270 epitopes. B: IHC for NeuN (upper panels) and histological staining with FJB (lower panels) of injected (left) and noninjected (right) hemispheres. C: IHC for MHCII for microgliosis (upper panel) and for GFAP for astrogliosis (lower panel) in injected (left) and non-injected (right) hemispheres. D: IHC with HT7 for human tau in AAV-Tau.255 injected mice (left panel) compared to AAV-TauP301L injected mice (right panel). Note the lack of neurodegeneration inflicted by Tau255 (panels A, B, C, D) and the different subcellular localization of Tau255 (panel D, left) versus Tau.P301L (panel D, right). Scale bars: A, C 0.5 mm; B 0.5 mm (upper panel) and 50 µm (lower panel); D 40 µm
(10.29 MB TIF)

Figure S6 Morphological and pathological aspects of Tau-mediated neurodegeneration. Intracerebral injection of 10E8 t.u. AAV-Tau.P301L vector in wild-type mice analyzed 3 weeks p.i. A. IHC for active caspase-3 and quantification of apoptotic cells in ipsilateral and contralateral hemispheres (mean, p<0.05, ANOVA single factor). Note the distribution of presumed apoptotic neurons (arrowheads) in regions that do not correlate with degenerating neurons. B. IHC for LC3 and Beclin as mediators of autophagy. Scale bar 40 µm. C. Histological and ultra-structural analysis of brain sections stained with toluidin-blue a–d: shrunken dark neurons (a,b red arrows) absent at contralateral side (c, d). e: vacuolization of cytoplasm (green arrow) and condensed chromatin (blue arrow) f: indentations of nuclei (red arrowheads). Scale bars: a–d 20 µm, e–f 2 µm. D. IHC with AT8 and AT270 reveal sporadic tangles, spheroids and axonal dilatations.
(9.31 MB TIF)

Figure S7 Lipofucsin in degenerating neurons. Autofluorescent lipofucsin-like deposits in brain of mice injected with 10E8 t.u. of AAV-Tau.P301L analyzed at different periods p.i. as indicated, with enlarged view at higher magnification (utmost right panel). The efficient elimination with a proprietary reagent is illustrated (panel marked “Autofluo-eliminator”).
(2.21 MB TIF)

Figure S8 Defects in AAV-Tau.P301L injected mice. Intracerebral injection of 10E8 t.u. of AAV-TauP301L in wild-type mice 3 weeks p.i. analyzed by IHC for tubulin, actin and synaptophysin in injected (left panels) and non-injected (right panels) hemispheres.
(9.50 MB TIF)

Table S1 Antibodies used in this study
(0.08 MB PDF)

Table S2 Markers tested by IHC on AAV-Tau.P301L mice.
(0.04 MB PDF)
