# Supplementary figures and images for: AAV-Tau Mediates Pyramidal Neurodegeneration by Cell-Cycle Re-Entry without Neurofibrillary Tangle Formation in Wild-Type Mice
Source: PLoS One. 2009 Oct 1;4(10):e7280. doi: 10.1371/journal.pone.0007280 (PMC2748684; doi:10.1371/journal.pone.0007280)

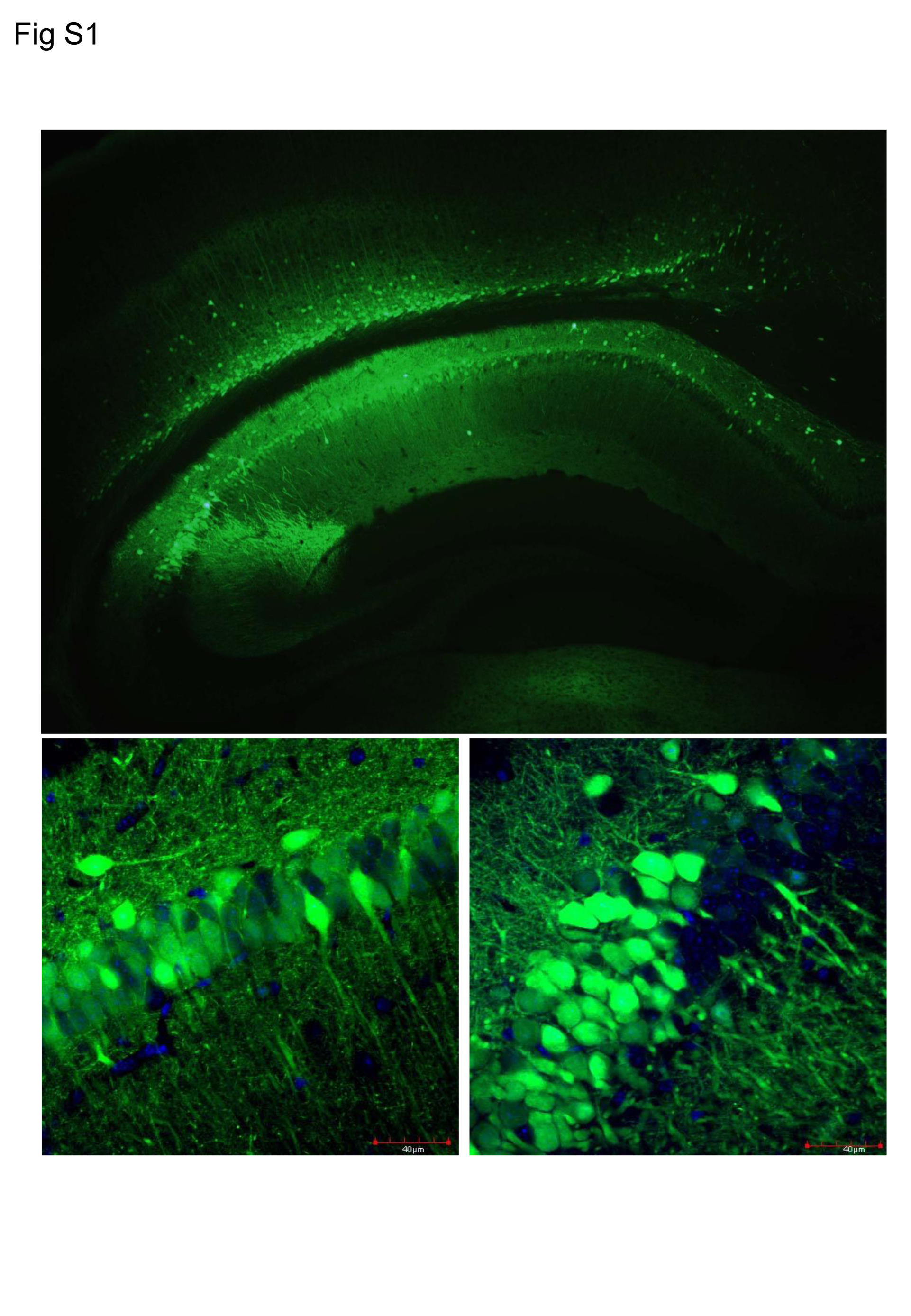

Supplement: Figure S1 — AAV1/2-mediated expression of EGFP. Intracerebral injection of 10E8 t.u. AAV-EGFP in wild-type mice analyzed 12 weeks months p.i. by fluorescence microscopy for EGFP in neurons in the hippocampal formation and in layers 5 and 6 of the cortex (upper panel). Confocal microscopy of CA1 (lower left panel) and CA2 (lower right panel) pyramidal neurons with EGFP expressed in soma and apical dendrites. Scale bar 40 µm. (8.88 MB TIF) [file pone.0007280.s001.tif]

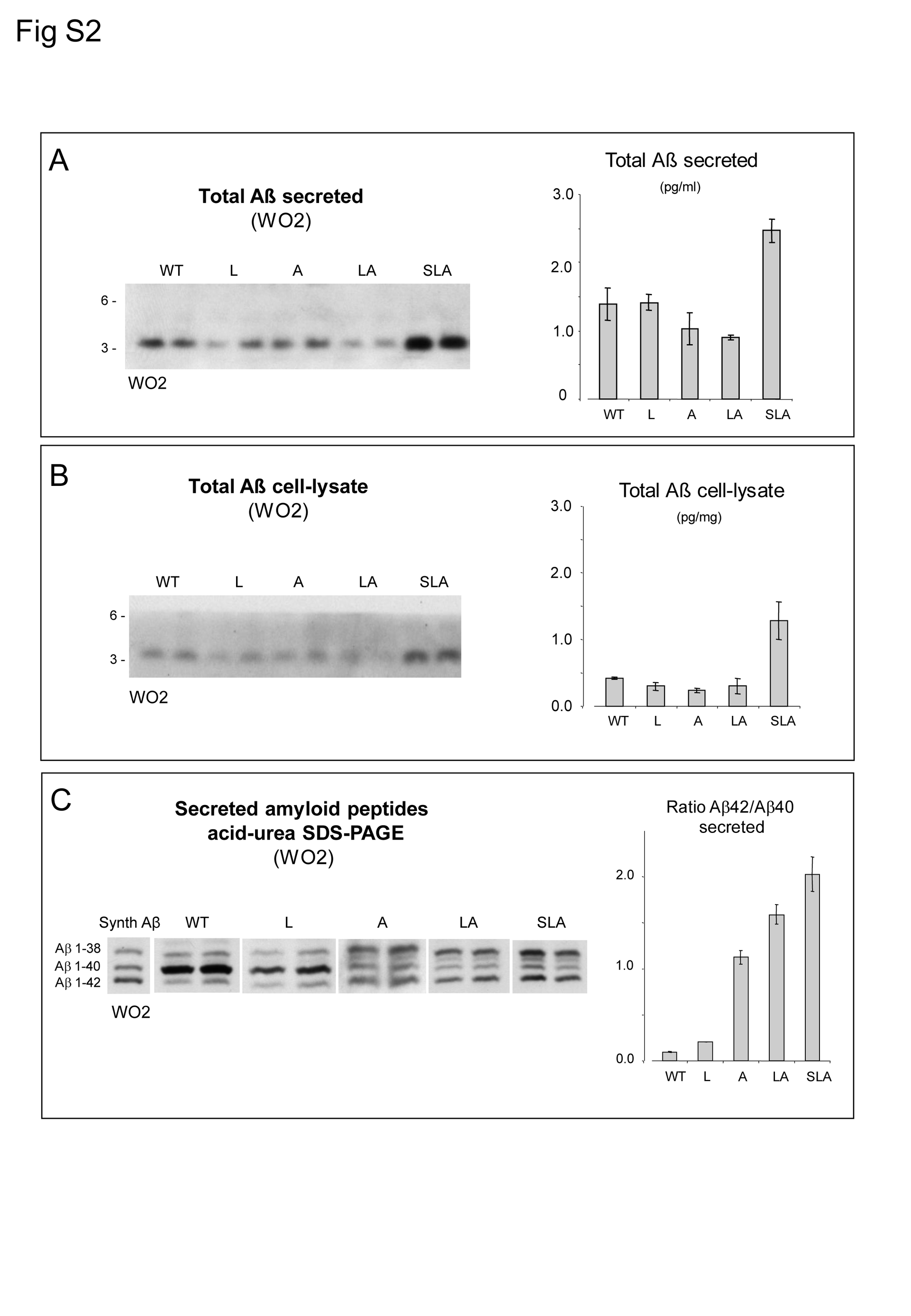

Supplement: Figure S2 — Effect of APP-mutations on generation of amyloid peptides in transfected N2a. Mouse neuroblastoma N2a cells transiently transfected with pcDNA3 vectors encoding human APP695 containing no, Swedish (K670M/N671L), London (V717I) or Austrian (T714I) mutations, alone and in the combinations indicated. Panels A:. cellular growth media were collected after 48 hours of culture, immunoprecipitated with Mab 6E10 and protein G-agarose beads before Western blotting with Mab WO2, after microwave heating of the filters, as recommended for the WO2 antibody. Panel B: Cell extracts analyzed directly by Western blotting with WO2. Panel C: acid-urea SDS-PAGE to separate amyloid peptides with synthetic amyloid peptides as standards (Aβ-mix). Quantification by densitometric scanning using synthetic peptides as standards. Note the highest ratio Aβ42/Aβ40 for APP.SLA triple mutant, which was used in the AAV-construct. (1.40 MB TIF) [file pone.0007280.s002.tif]

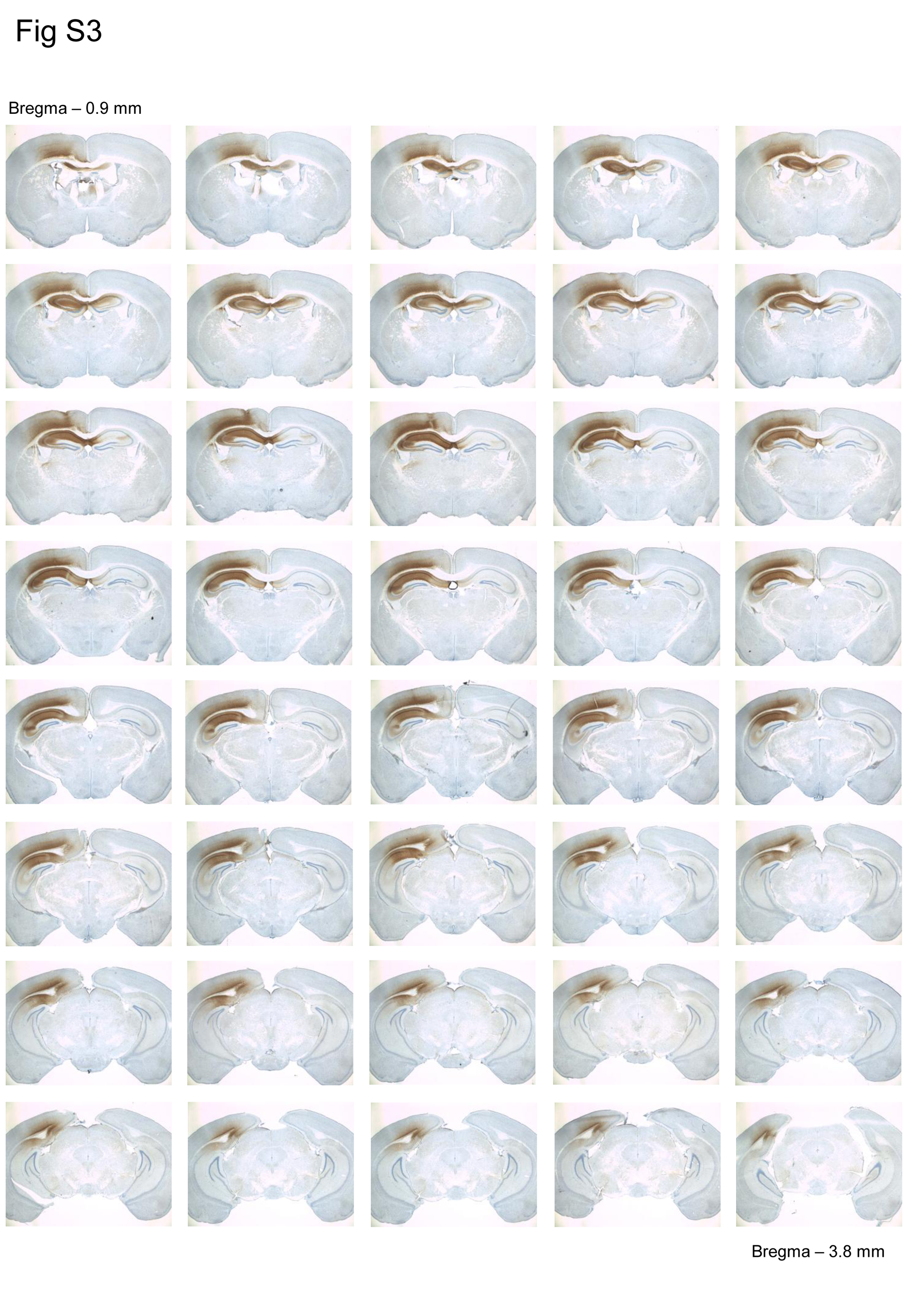

Supplement: Figure S3 — Distribution of human tau following AAV-Tau.P301L injection. Compilation of 40 sections (each 40 µm) spaced each about 3–4 sections apart throughout the brain of a wildtype mouse injected with 10E8 t.u. AAV-TauP301L and analyzed at 1.5 weeks p.i. for human protein tau by IHC with Mab HT7. (10.05 MB TIF) [file pone.0007280.s003.tif]

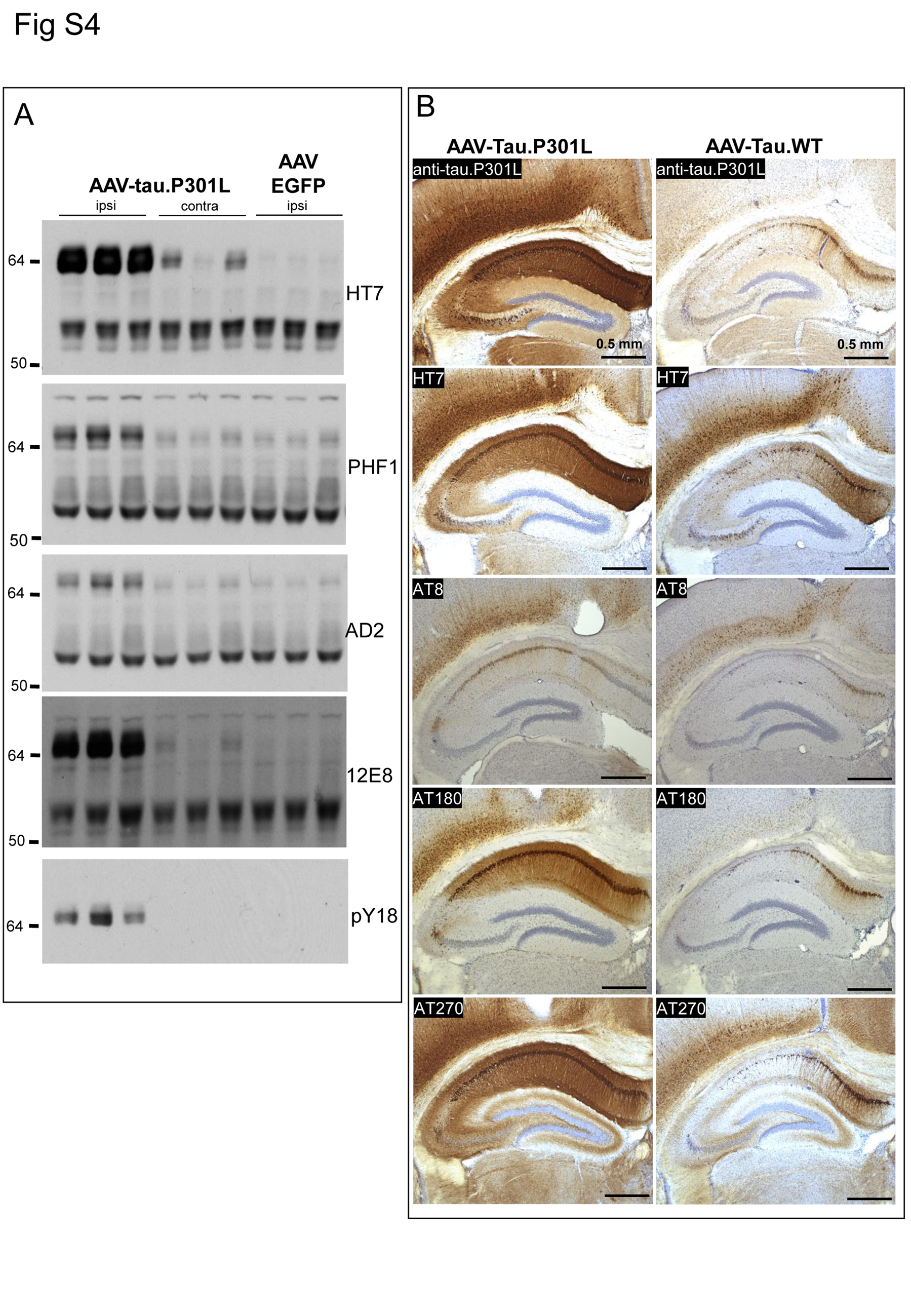

Supplement: Figure S4 — Comparison of wild-type and mutant Tau. Intracerebral injection of 10E8 t.u. of the indicated AAV-vectors in wild-type mice analyzed 1.5 week (panel A) and 3 weeks p.i. (panel B) with indicated antibodies in Western blotting and IHC, respectively. Note some minor cross-reaction of the polyclonal antibody against Tau.P301L (ref. 46) with wild-type Tau in IHC (right upper panel). (10.17 MB TIF) [file pone.0007280.s004.tif]

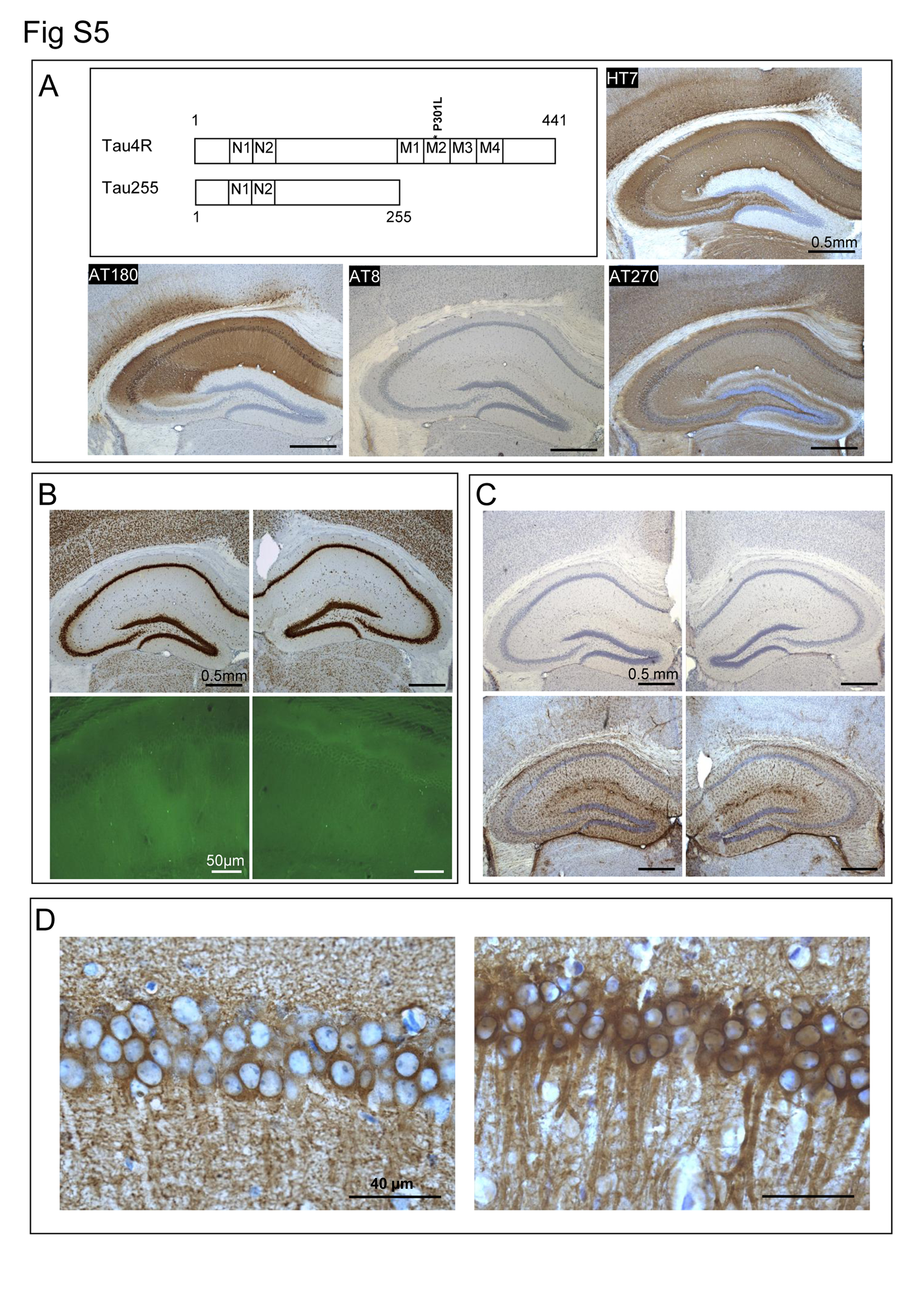

Supplement: Figure S5 — Protein Tau255 lacking microtubuli binding domains is not neurotoxic. Intracerebral injection of 10E8 t.u. AAV-Tau255 vector in wild-type mice (n = 8) analyzed 3 weeks p.i. A: representation of Tau.255 and Tau4R constructs and representative IHC for human Tau with HT7, AT180, AT8, AT270. Note that Tau.255 lacks phosphorylation at AT8 and AT270 epitopes. B: IHC for NeuN (upper panels) and histological staining with FJB (lower panels) of injected (left) and noninjected (right) hemispheres. C: IHC for MHCII for microgliosis (upper panel) and for GFAP for astrogliosis (lower panel) in injected (left) and non-injected (right) hemispheres. D: IHC with HT7 for human tau in AAV-Tau.255 injected mice (left panel) compared to AAV-TauP301L injected mice (right panel). Note the lack of neurodegeneration inflicted by Tau255 (panels A, B, C, D) and the different subcellular localization of Tau255 (panel D, left) versus Tau.P301L (panel D, right). Scale bars: A, C 0.5 mm; B 0.5 mm (upper panel) and 50 µm (lower panel); D 40 µm (10.29 MB TIF) [file pone.0007280.s005.tif]

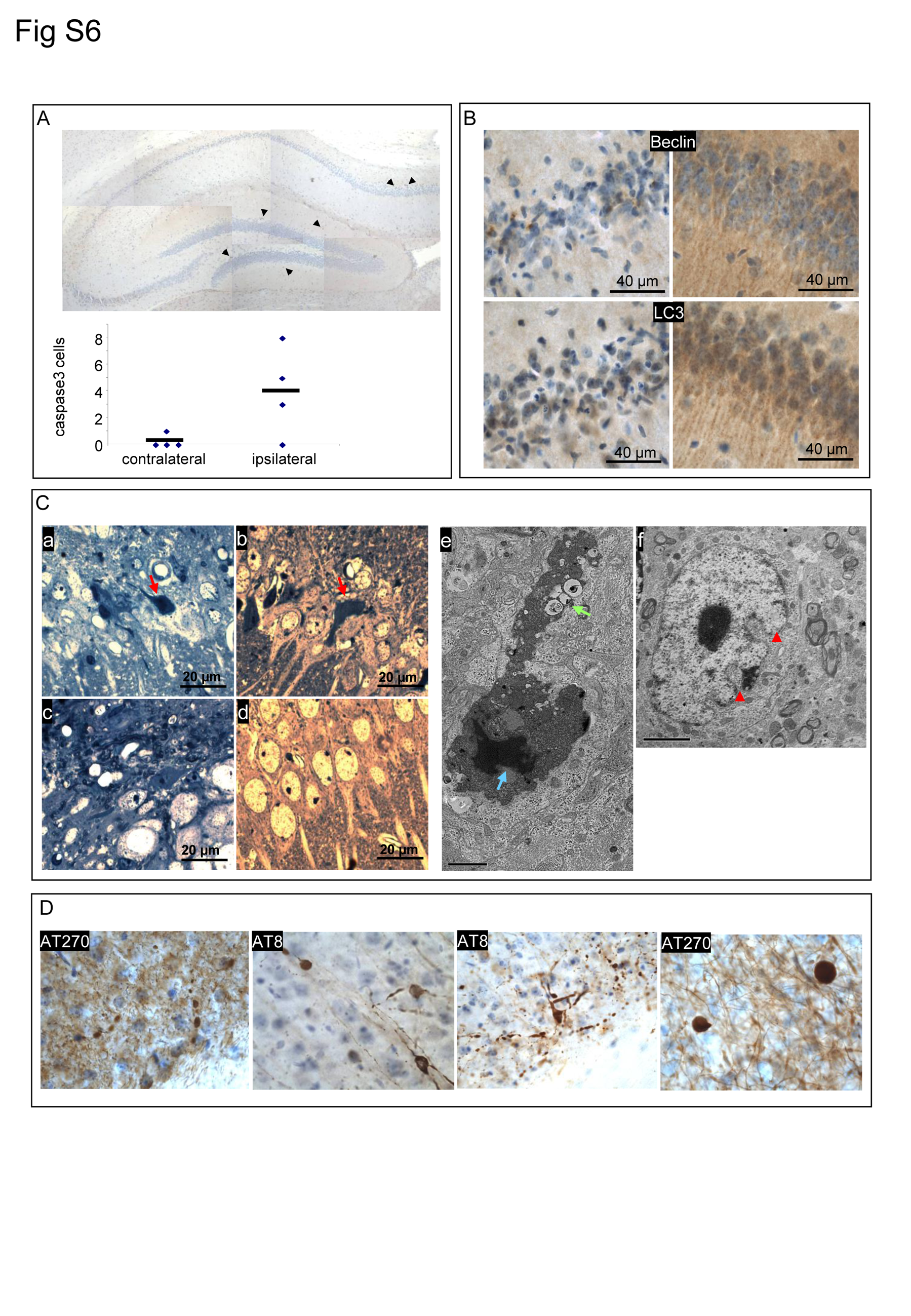

Supplement: Figure S6 — Morphological and pathological aspects of Tau-mediated neurodegeneration. Intracerebral injection of 10E8 t.u. AAV-Tau.P301L vector in wild-type mice analyzed 3 weeks p.i. A. IHC for active caspase-3 and quantification of apoptotic cells in ipsilateral and contralateral hemispheres (mean, p<0.05, ANOVA single factor). Note the distribution of presumed apoptotic neurons (arrowheads) in regions that do not correlate with degenerating neurons. B. IHC for LC3 and Beclin as mediators of autophagy. Scale bar 40 µm. C. Histological and ultra-structural analysis of brain sections stained with toluidin-blue a–d: shrunken dark neurons (a,b red arrows) absent at contralateral side (c, d). e: vacuolization of cytoplasm (green arrow) and condensed chromatin (blue arrow) f: indentations of nuclei (red arrowheads). Scale bars: a–d 20 µm, e–f 2 µm. D. IHC with AT8 and AT270 reveal sporadic tangles, spheroids and axonal dilatations. (9.31 MB TIF) [file pone.0007280.s006.tif]

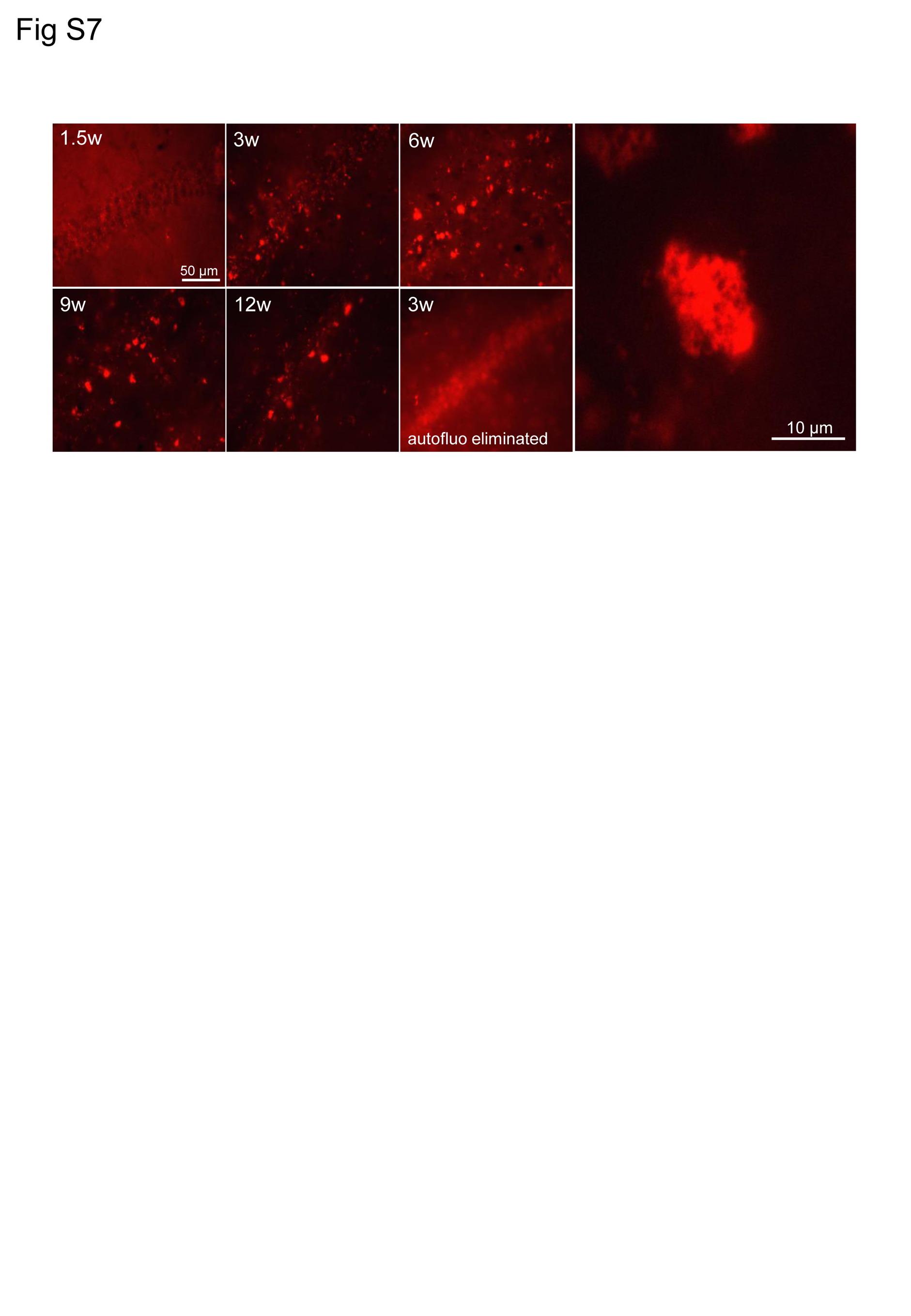

Supplement: Figure S7 — Lipofucsin in degenerating neurons. Autofluorescent lipofucsin-like deposits in brain of mice injected with 10E8 t.u. of AAV-Tau.P301L analyzed at different periods p.i. as indicated, with enlarged view at higher magnification (utmost right panel). The efficient elimination with a proprietary reagent is illustrated (panel marked “Autofluo-eliminator”). (2.21 MB TIF) [file pone.0007280.s007.tif]

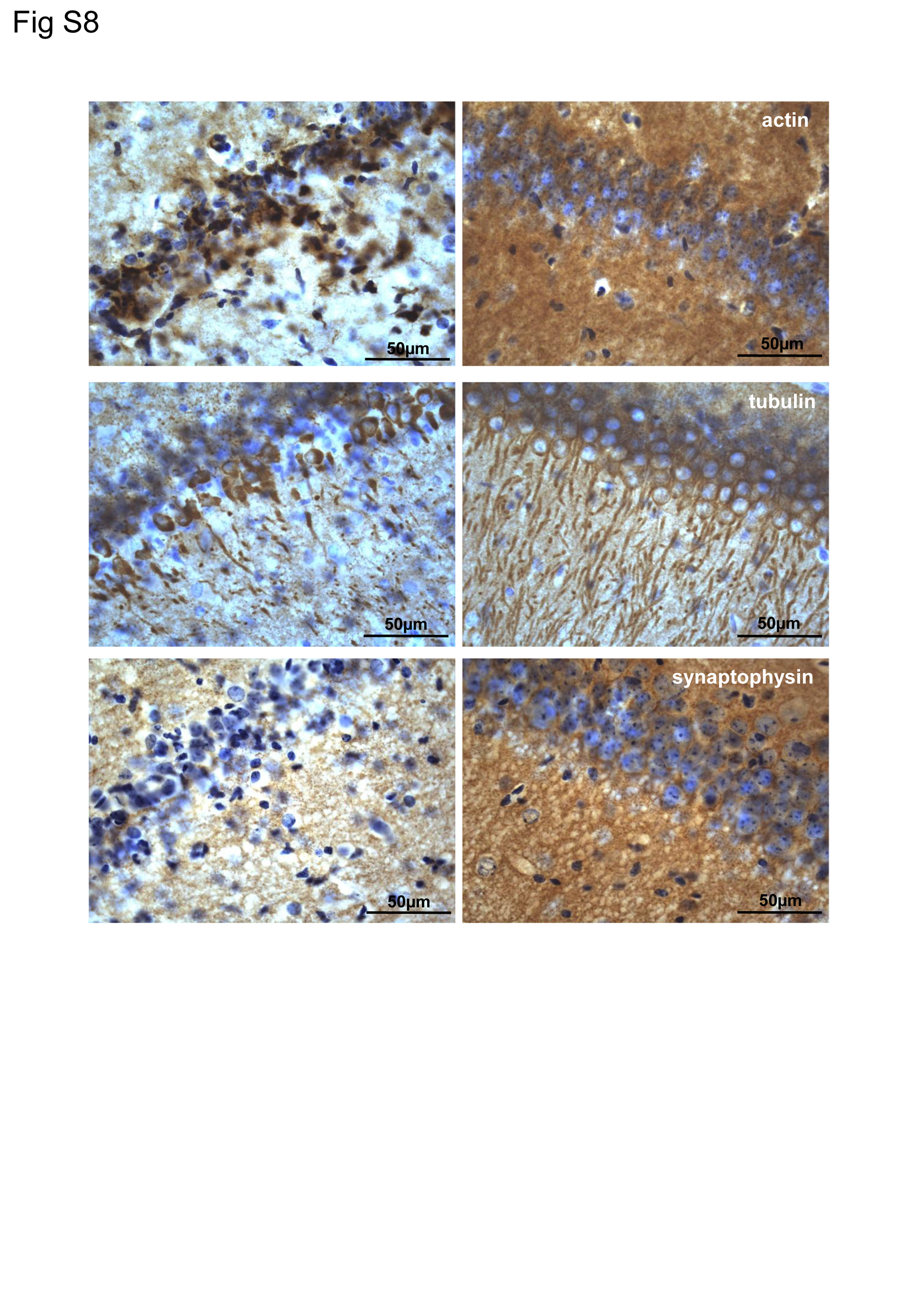

Supplement: Figure S8 — Defects in AAV-Tau.P301L injected mice. Intracerebral injection of 10E8 t.u. of AAV-TauP301L in wild-type mice 3 weeks p.i. analyzed by IHC for tubulin, actin and synaptophysin in injected (left panels) and non-injected (right panels) hemispheres. (9.50 MB TIF) [file pone.0007280.s008.tif]
