# Supplementary material for: AAV-Tau Mediates Pyramidal Neurodegeneration by Cell-Cycle Re-Entry without Neurofibrillary Tangle Formation in Wild-Type Mice
Source: PLoS One. 2009 Oct 1;4(10):e7280. doi: 10.1371/journal.pone.0007280 (PMC2748684; doi:10.1371/journal.pone.0007280)
Supplement: Table S1 — Antibodies used in this study (0.08 MB PDF) [file pone.0007280.s009.pdf]

Table S1. Antibodies used in this study

Antibody	Type	Specificity / Epitope	Label	Host	Concentration or dilution	Supplier
**Protein tau**						
Tau5	Monoclonal	Most species tau	-	Mouse	WB: 0.25 μg/ml	Pharmingen
HT7	Monoclonal	Human tau	-	Mouse	WB: 0.2 μg/ml	Pierce
HT7	Monoclonal	Human tau	biotin	Mouse	IHC: 0.17 μg/ml	Innogenetics
Tau.P301L	Polyclonal	P301L tau	-	Rabbit	IHC: 1/10000	P. Heutink
AT8	Monoclonal	P-Ser202/P-Thr205	biotin	Mouse	IHC: 0.56 μg/ml	Innogenetics
AT270	Monoclonal	P-Thr181	biotin	Mouse	IHC: 0.16 μg/ml	Innogenetics
AT270	Monoclonal	Endogenous tau P-Thr181	biotin	Mouse	IHC: 1.6 μg/ml	Innogenetics
AT180	Monoclonal	P-Thr231	biotin	Mouse	IHC: 0.18 μg/ml	Innogenetics
AT180	Monoclonal	Endogenous tau P-Thr231	biotin	Mouse	IHC: 1.8 μg/ml	Innogenetics
AD2	Monoclonal	P-Ser396/P-Thr404	-	Mouse	WB: 0.4 μg/ml	BioRad
PHF1	Monoclonal	P-Ser396/P-Thr404	-	Mouse	WB: 1/250	P. Davies (New York)
12E8	Monoclonal	P-Ser262	-	Mouse	WB: 3.5 μg/ml	Elan
pY18	Polyclonal	P-Tyr18	-	Rabbit	WB: 1/1000	G. Lee (Iowa)
**Protein APP**						
B10.4	Polyclonal	C-terminal APP	-	Rabbit	WB: 1/5000	Home made
WO2	Monoclonal	aa 4-10 of Aβ	-	Mouse	WB: 1/1000	The Genetics Company
3D6	Monoclonal	aa 1-5 of Aβ	biotin	Mouse	IHC: 0.5 μg/ml	Innogenetics
6E10	Monoclonal	aa 1-17 of Aβ	biotin	Mouse	IHC: 1 μg/ml	Signet
**Inflammation**						
GFAP	Polyclonal	Astrocytes	-	Rabbit	IHC: 0.06 μg/ml	Dako
MHCII	Polyclonal	Activated microglia	-	Rat	IHC: 0.05 μg/ml	Pharmingen
**Cell cycle**						
Ki67	Polyclonal	1086 bp Ki67 cDNA	-	Rabbit	IHC: 1/10000	Novocastra
PCNA	Polyclonal	aa 1-261	-	Rabbit	IHC: 0.04 μg/ml	Santa Cruz
p27KIP1	Polyclonal	C-terminal	-	Rabbit	IHC: 2 μg/ml	Santa Cruz
Phospho-pRb	Polyclonal	P-Ser807/811	-	Rabbit	IHC: 1/500	Cell Signaling
Cyclin B1	Polyclonal	C-terminal	-	Rabbit	IHC: 0.4 μg/ml	Santa Cruz
Cyclin D2	Polyclonal	aa 1-289	-	Rabbit	IHC: 0.4 μg/ml	Santa Cruz
**Cytoskeleton**						
Actin	Polyclonal	C-terminal	-	Rabbit	IHC: 1/10000	Sigma
Tubulin	Polyclonal	Human α/β tubulin	-	Rabbit	IHC: 1/1000	Cell Signaling
Synaptohysin	Polyclonal	Human synaptohysin	-	Rabbit	IHC: 1/10000	Dako
**Other**						
NeuN	Monoclonal	Neuronal nuclei	biotin	Mouse	IHC: 1 μg/ml	Chemicon
LC3	Polyclonal	aa 50-150	-	Rabbit	IHC: 1/10000	ABR
Beclin	Polyclonal	aa 329-345	-	Rabbit	IHC: 0.13 μg/ml	Abcam
Casapse-3	Polyclonal	Activated caspase-3	-	Rabbit	IHC: 1/500	Cell Signaling
pp38 MAPK	Polyclonal	pThr180/pTyr182	-	Rabbit	IHC: 1/100	New England Biolabs
pSAPK/JNK	Polyclonal	pThr183/pTyr185	-	Rabbit	IHC: 1/100	New England Biolabs
p-p44/42 MAPK (Erk1/2)	Polyclonal	pThr202/pTyr204	-	Rabbit	IHC: 1/100	New England Biolabs
