# Supplementary material for: AAV-Tau Mediates Pyramidal Neurodegeneration by Cell-Cycle Re-Entry without Neurofibrillary Tangle Formation in Wild-Type Mice
Source: PLoS One. 2009 Oct 1;4(10):e7280. doi: 10.1371/journal.pone.0007280 (PMC2748684; doi:10.1371/journal.pone.0007280)
Supplement: Table S2 — Markers tested by IHC on AAV-Tau.P301L mice. (0.04 MB PDF) [file pone.0007280.s010.pdf]

Table S2. Markers tested by IHC on AAV-Tau.P301L mice

Marker protein	Function / action	Changes in AAV-Tau.P301L	
		Neurons	Glia
**Cell cycle**			
Cyclin B1	G2 phase progression	↑	
Cyclin D2	G1 phase progression	↑	
Cyclin D3	G1 phase progression		↑
Cyclin A	S phase progression		↑
p27KIP1	Cdk inhibitor	↓	
Cdk2	G1-S transition	-	
p57KIP2	Cdk inhibitor	↓	↑
phospho-Rb	E2F1 inhibitor	↑	
Ki67	G1, S, G2, M phase	-	
PCNA	DNA Pol processivity factor	↑	
PIN1	Peptidyl-prolyl isomerase		↑
**Inflammation**			
GFAP	Activated of astroglia		↑
MHCII	Major histocompatibility complex		↑
**Apoptosis**			
Caspase-3 cleaved	Cysteine-aspartic acid protease	↑	
**Autophagy**			
Beclin	Autophagosome formation	↓	
LC3	Autophagosome formation	↓	
**Cytoskeleton**			
Actin	Cytoarchitecture	↓	
Tubulin	Microtubules constituent	↓	
Synaptophysin	Synaptic vesicle protein	↓	
**Stress**			
nNOS	Neuronal Nitric Oxide Synthase	-	
Nitrotyrosine	Cell damage and inflammation		↑
**Protein quality control**			
GRP78	Unfolded protein response		↑
**Signalling**			
p38 MAPK (T180/Y182)	Mitogen-activated protein kinase	↑	
p44/42 MAPK (Erk1/2) T202/Y204	Extracellular signal-regulated kinase		↑
pSAPK/JNK (T183/Y185)	Stress-activated protein kinase	-	
P-Akt Ser473	Ser/Thr kinase	↓	
P-Akt Thr308	Ser/Thr kinase	↑	
P-GSK3a S21	Ser/Thr kinase		↑
P-GSK3b S9	Ser/Thr kinase	↑	
P-GSK3a/b S21/9	Ser/Thr kinase	-	
Cdk5	Cyclin dependent kinase	↓	
P-Cdk5 S159	Cyclin dependent kinase	↑	
P-GSK3a/b Y279/216	Ser/Thr kinase	-	
P-ATM S1981	DNA damage checkpoint	-	
**Others**			
P-H2AX S139	DNA Double-strand breaks	↑	
Cathepsin B	Serine protesae	↓	
Ubiquitin	Protein degradation	↓	
Dkk1	Wnt signaling inhibitor	-	
Doublecortin	Neurogenesis	-	
b-catenin	Transcription coregulator	↓	
c-fos	Transcription factor	-	
p35	Cdk5 activator	↓	↑
P-CREB	Transcription factor	↑	

↑, increase; ↓, decrease; - , no change
